# Jian-Ti-Kang-Yi decoction alleviates poly(I:C)-induced pneumonia by inhibiting inflammatory response, reducing oxidative stress, and modulating host metabolism

**DOI:** 10.3389/fphar.2022.979400

**Published:** 2022-09-06

**Authors:** Huantian Cui, Yuming Wang, Bolun Yu, Yulin Wu, Gaijun Zhang, Junli Guo, Junyu Luo, Qin Li, Xiaojuan Li, Wenju He, Weibo Wen, Jiabao Liao, Dongqiang Wang

**Affiliations:** ^1^ Shandong Provincial Key Laboratory of Animal Cell and Developmental Biology, School of Life Sciences, Shandong University, Qingdao, China; ^2^ Graduate School, Tianjin University of Traditional Chinese Medicine, Tianjin, China; ^3^ Tianjin Medical University Cancer Institute and Hospital, Tianjin, China; ^4^ Hebei Hospital of Traditional Chinese Medicine, Hebei, China; ^5^ Yunnan Provincial Hospital of Chinese Medicine, Kunming, Yunnan, China; ^6^ Yunnan University of Traditional Chinese Medicine, Kunming, Yunnan, China; ^7^ Jiaxing Hospital of Traditional Chinese Medicine, Jiaxing, Zhejiang, China; ^8^ Tianjin First Central Hospital, Tianjin, China

**Keywords:** poly(I:C)-induced pneumonia, Jian-Ti-Kang-Yi decoction, antiinflammatory effect, anti-oxidative effect, untargeted metabolomics

## Abstract

Jian-Ti-Kang-Yi decoction (JTKY) is widely used in the treatment of COVID-19. However, the protective mechanisms of JTKY against pneumonia remain unknown. In this study, polyinosinic-polycytidylic acid (poly(I:C)), a mimic of viral dsRNA, was used to induce pneumonia in mice; the therapeutic effects of JTKY on poly(I:C)-induced pneumonia model mice were evaluated. In addition, the anti-inflammatory and anti-oxidative potentials of JTKY were also investigated. Lastly, the metabolic regulatory effects of JTKY in poly(I:C)-induced pneumonia model mice were studied using untargeted metabolomics. Our results showed that JTKY treatment decreased the wet-to-dry ratio in the lung tissue, total protein concentration, and total cell count of the bronchoalveolar lavage fluid (BALF). Hematoxylin and Eosin (HE) and Masson staining indicated that the JTKY treatment alleviated the pathological changes and decreased the fibrotic contents in the lungs. JTKY treatment also decreased the expression of pro-inflammatory cytokines [interleukin (IL)-1β, IL-6, and tumor necrosis factor-alpha (TNF-α)] and increased the levels of immunomodulatory cytokines (IL-4 and IL-10) in the BALF and serum. Flow cytometry analysis showed that the JTKY treatment lowered the ratio of CD86^+^/CD206^+^ macrophages in the BALF, decreased inducible nitric oxide synthase (iNOS) level, and increased arginase 1 (Arg-1) level in lung. JTKY also lowered CD11b^+^Ly6G^+^ neutrophils in BALF and decreased myeloperoxidase (MPO) activity in lung. Moreover, it also elevated superoxide dismutase (SOD) and glutathione peroxidase (GSH-Px) activities and decreased methane dicarboxylic aldehyde (MDA) level in lung. Untargeted metabolomic analysis showed that the JTKY treatment could affect 19 metabolites in lung, such as L-adrenaline, L-asparagine, ornithine, and alpha-ketoglutaric acid. These metabolites are associated with the synthesis and degradation of ketone bodies, butanoate, alanine, aspartate, and glutamate metabolism, and tricarboxylic acid (TCA) cycle processes. In conclusion, our study demonstrated that treatment with JTKY ameliorated poly(I:C)-induced pneumonia. The mechanism of action of JTKY may be associated with the inhibition of the inflammatory response, the reduction of oxidative stress, and the regulation of the synthesis and degradation of ketone bodies, TCA cycle, and metabolism of alanine, aspartate, glutamate, and butanoate processes in lung.

## Introduction

Viral pneumonia is an infection of the upper respiratory tract caused by viruses; it spreads downwards and infects the lungs, resulting in pulmonary ventilation disorders. Viral pneumonia poses a significant threat to global public health owing to the outbreak and spread of COVID-19 (Ning et al., 2022; Tiecco et al., 2022). Viruses exhibit a high degree of genetic variability and complex pathogenic mechanisms, which impede the development of vaccines and antiviral drugs. As a result, the development of safe and effective drug therapy for viral pneumonia has the potential to significantly reduce its adverse effects.

Numerous studies have revealed the beneficial effects of traditional Chinese medicine (TCM) on viral pneumonia (Cui H. T. et al., 2020; Sumathi et al., 2021). Early application of the Qing-Fei-Pai-Du decoction can significantly reduce hospital stay and improve clinical symptoms in patients with COVID-19 (Shi et al., 2020; Zhang et al., 2020); a meta-analysis concluded that Lian-Hua-Qing-Wen capsules used in combination with conventional treatment had a clinical efficacy of 95% in patients with COVID-19, improving chest computed tomography scan manifestations and reducing the incidence of dialysis (Liu M. et al., 2021). Additionally, a retrospective study discovered that the He-Jie-Shen-Shi decoction was effective in decreasing the time required for the nucleic acid amplification test to become negative and alleviating clinical symptoms, such as fever and cough, in patients with severe COVID-19 (Hu et al., 2021). Clarifying the mechanism of action of TCM in the treatment of viral pneumonia can help establish a scientific foundation for it’s clinical application.

Cytokine storms are an exaggerated immune response to external stimuli, such as viruses (Kim et al., 2021), characterized by a high level of immune cell activation and a high production of pro-inflammatory cytokines and chemical mediators. Viral infections result in the release of chemokines in lung tissue, which stimulates the migration of various immune cells, such as macrophages and neutrophils, to the lungs, and can result in the over-activation of immune cells, causing the production of a large number of cytokines, such as TNF-α, IL-1β, and IL-6 (Soy et al., 2020). These pro-inflammatory cytokines can stimulate the activation of additional immune cells and damage the alveolar-capillary network, resulting in acute respiratory distress syndrome and multiple organ system failure (Iannaccone et al., 2020).

The Jian-Ti-Kang-Yi decoction (JTKY) contains *Astragalus mongholicus* Bunge, *Atractylodes macrocephala* Koidz., *Saposhnikovia divaricata* (Turcz. ex Ledeb.) Schischk., *Glehnia littoralis* (A. Gray) F.Schmidt ex Miq., *Ophiopogon japonicus* (Thunb.) Ker Gawl, *Ganoderma lucidum* (Curtis) P. Karst., *Platycodon grandiflorus* (Jacq.) A. DC., *Forsythia suspensa* (Thunb.) Vahl, *Perilla frutescens* (L.) Britton, *Agastache rugosa* (Fisch. & C. A. Mey.) Kuntze, *Lonicera japonica* Thunb., and *Glycyrrhiza uralensis* Fisch. ex DC., which is widely used in the treatment of COVID-19 patients in Yunan, China, and other regions. However, the mechanism by which JTKY treats pneumonia remains unclear. In this study, we established a pneumonia model *via* the intratracheal instillation of polyinosinic-polycytidylic acid [poly(I:C)], a mimic of viral dsRNA. First, we examined the therapeutic effects of JTKY in poly(I:C)-induced pneumonia model mice. Thereafter, we examined the effects of JTKY on inflammatory cytokine storms. Finally, we investigated the mechanism of action of JTKY using an untargeted metabolomic approach.

## Materials and methods

### Reagents

Poly(I:C) was purchased from Yuanye Bio-Technology Co., Ltd. (Shanghai, China). Dexamethasone (DXM) was purchased from Solarbio Biotechnology Co. Ltd. (Beijing, China). Total protein, superoxide dismutase (SOD), methane dicarboxylic aldehyde (MDA), glutathione peroxidase (GSH-Px), inducible nitric oxide synthase (iNOS), and myeloperoxidase (MPO) assay kits were obtained from the Nanjing Jiancheng Biological Engineering Institute (Nanjing, China). Enzyme-linked immunosorbent assay (ELISA) kits of mouse arginase 1 (Arg-1), and interleukin (IL)-1β, IL-6, IL-10, and IL-4 were purchased from Multi Science Biotechnology Co. Ltd. (Hangzhou, China). ELISA kits of mouse tumor necrosis factor-alpha (TNF-α) were purchased from Shanghai BlueGene Biotech Co., Ltd. (Shanghai, China). PE anti-mouse CD86, APC anti-mouse CD206, APC anti-mouse Ly6G, and PE anti-mouse CD11b antibodies were purchased from BioLegend, Inc. (Beijing, China).

### Experimental animals

Male C57BL/6 mice (weight, 20 ± 2 g) were provided by Beijing Huafukang Biotechnology Co., Ltd. The mice were housed in a specific pathogen free (SPF)-grade clean environment; there were five animals per cage with free access to food and water and a temperature of 22°C ± 2°C, humidity of 50% ± 15%, and day/night cycle. All animal experiments were conducted in accordance with the National Institutes of Health Guidelines for the Care and Use of Laboratory Animals, and all procedures were approved by the Animal Medicine and Animal Protection Ethics Committee of Yunnan University of Traditional Chinese Medicine. All experimental procedures were conducted in strict accordance with the regulations and requirements of the Animal Laboratory.

### Pneumonia model generation using a transtracheal injection of poly(I:C)

After administering isoflurane for basic anesthesia, the incisors and limbs of the mice were fixed in the supine position, on a mouse plate that was tilted at 60° and placed on a clean bench, with the head elevated and the tail tucked low. The neck was completely exposed and disinfected; the trachea was gradually visible, and the drug was aspirated using a 1 ml syringe. The needle was stabbed obliquely in the centripetal direction into the trachea using the left hand to secure it. Additionally, upon sensing poking into space, a portion of the needle was pushed horizontally forward. If the needle experienced the presence of air, or no resistance upon withdrawal, it was considered to be in the trachea. A 5 mg/kg solution of poly(I:C) [dissolved in 50 μl of phosphate-buffered saline (PBS)] was slowly instilled into the trachea; a small amount of air was injected at the end to ensure that the entire solution reached the area. Following intratracheal instillation, the plate was immediately raised and gently shaken from side to side to ensure that the drug was evenly distributed throughout the lungs. When the narcosis wore off and the mice resumed spontaneous respiration and were awake, they were returned to their cages, and the model generation was complete.

### Preparation of Jian-Ti-Kang-Yi decoction

A total of 15 g of *Astragalus mongholicus* Bunge, 12 g of *Atractylodes macrocephala* Koidz., 12 g of *Saposhnikovia divaricata* (Turcz. ex Ledeb.) Schischk., 15 g of *Glehnia littoralis* (A. Gray) F. Schmidt ex Miq., 12 g of *Ophiopogon japonicus* (Thunb.) Ker Gawl., 12 g of *Ganoderma lucidum* (Curtis) P. Karst., 12 g of *Platycodon grandiflorus* (Jacq.) A. DC., 12 g of *Forsythia suspensa* (Thunb.) Vahl, 12 g of *Perilla frutescens* (L.) Britton, 12 g of *Agastache rugosa* (Fisch. & C. A. Mey.) Kuntze, 12 g of *Lonicera japonica* Thunb., and 9 g of *Glycyrrhiza uralensis* Fisch. ex DC. were obtained. Detailed information of each herb used in JTKY is shown in Table 1. The herbs were identified by Dr. Qin Li from the Yunnan Provincial Hospital of Chinese Medicine; voucher specimens were deposited in the pharmacy department of the hospital. The herbs were first combined with 1176 ml of water (eight times the amount of raw herb), soaked for 2 h, decocted for 60 min, and then filtered. The filtrate was stored separately, and the dregs were then mixed with 882 ml of water (six times the amount of raw herb) for the second extraction. About 300 ml of filtrates each time were obtained and combined. Then, the combined filtrates (600 ml) were concentrated to about 300 ml (490 mg of crude herb/ml). Considering the capacity of mice stomach, the obtained JTKY extraction was then evaporated to 2.21 g of crude herb/ml and 4.42 g of crude herb/ml, respectively. It was stored at 4°C. The production license of JTKY was shown in Supplementary Figure S1.

**TABLE 1:** Detailed information of herbs in JTKY.

Chinese name	Latin name	Batch no.	Production company
Huangqi	*Astragalus mongholicus* Bunge	YP20211101J	Traditional Chinese Medicine Resources Co., Ltd. of Yunnan Baiyao Group
Baizhu	*Atractylodes macrocephala* Koidz.	211200461	Chengdu Kangmei Pharmaceutical Co., Ltd.
Fangfeng	*Saposhnikovia divaricata* (Turcz. ex Ledeb.) Schischk.	20200501	Yunnan Jianantang Biotechnology Co., Ltd.
Beishashen	*Glehnia littoralis* (A. Gray) F.Schmidt ex Miq.	2111132	Sichuan Xinhehua Herbal Pieces Co., Ltd.
Maidong	*Ophiopogon japonicus* (Thunb.) Ker Gawl.	190806	Anhui Zhiliang Herbal Pieces Co., Ltd.
Lingzhi	*Ganoderma lucidum* (Curtis) P. Karst.	2109021	Yunnan Hehe Herbal Pieces Co., Ltd.
Jiegeng	*Platycodon grandiflorus* (Jacq.) A. DC.	2105002	Yunnan Hehe Herbal Pieces Co., Ltd.
Lianqiao	*Forsythia suspensa* (Thunb.) Vahl	20072103	Yunnan Jingtian Biotechnology Co., Ltd.
Zisu	*Perilla frutescens* (L.) Britton	190806	Anhui Zhiliang Herbal Pieces Co., Ltd.
Huoxiang	*Agastache rugosa* (Fisch. & C. A. Mey.) Kuntze	20200501	Yunnan Jianantang Biotechnology Co., Ltd.
Jinyinhua	*Lonicera japonica* Thunb.	20211201	Guilin Zhongnan Pharmaceutical Co., Ltd.
Gancao	*Glycyrrhiza uralensis* Fisch. ex DC.	211101	Yunnan Daodi Herbal Pieces Co., Ltd.

Identification of the main compounds in JTKY was performed using ultra-performance liquid chromatography (UPLC; ACQUITY UPLC^®^, United States) coupled with a Xevo G2 quadrupole-time-of-flight (Q-TOF) mass spectrometer (MS; Waters Corp., Milford, MA, United States) based on our previously established protocol (Xie et al., 2021, Supplementary Material). The experimental conditions for the UPLC-Q/TOF-MS are shown in the Supplementary Material.

### Experimental grouping and drug administration protocol

Fifty mice were used for this study. After 1 week of adaptive feeding, they were randomly assigned to one of five groups: control, poly(I:C), DXM, JTKY low-dose, or JTKY high-dose groups. The poly(I:C), DXM, JTKY low-dose, and JTKY high-dose groups received a poly(I:C) injection to generate pneumonia mouse model, while the control group received 50 μl of PBS *via* an intratracheal injection. Following successful model generation, mice in the control and poly(I:C) groups were orally treated with saline (0.1 ml/10 g) once every 12 h. The DXM group received 5 mg/kg of DXM intraperitoneally. The JTKY low- and high-dose groups received oral treatments of 2.21 g/ml and 4.42 g/ml of JTKY extraction, respectively, twice within 24 h. The gavage amount of JTKY was 0.1 ml/10 g. Clinically, the dosage of JTKY for the treatment of pneumonia in adults (60 kg) is 147 g (the total raw materials) twice a day, indicating a dose of 2.45 g/kg twice a day. The dosage of JTKY for mice was calculated using the animal dose conversion formula based on the daily human dose, with a conversion coefficient of 9. The amount used for the low-dose JTKY mice group represents the human equivalent dose using the following formula: low-dose JTKY = 147 g (the total raw materials)/60 kg (human weight) × 9 (conversion coefficient).

Twenty-four hours after the JTKY treatment, the mice were anesthetized by an intraperitoneal injection of Nembutal (50 mg/kg), and their blood was collected from the retrobulbar plexus. The blood samples were centrifuged at 3,000 rpm/min for 15 min to obtain the serum.

### Collection of bronchoalveolar lavage fluid

The thoracic cavity was opened, and the cervical trachea was exposed layer by layer. A Lanz incision was made in the trachea, and a lavage tube was inserted into the lower end of the right main bronchus. The trachea and mouse lavage tubes were sutured together. The hilum of the left lung was then firmly sutured to ensure that it was airtight. One milliliter of saline was withdrawn using a syringe and connected to the lavage tube ligated in the cervical trachea; saline was slowly injected into the mice’s right lung *via* the syringe. Saline was left in the alveoli for 15–30 s before being gently sucked back to obtain the BALF; during the lavage, the saline was monitored closely for exudation. The injection and withdrawal process was repeated three times, and each mouse produced approximately 2.5 ml of BALF.

### Measurement of wet-to-dry weight ratio of lungs

At the end of the BALF collection in each group, the left lungs of the mice that had not been lavaged were collected. The wet mass (W) of the left lung was first weighed, and then dried for 48-h at a constant temperature of 80°C until the lung weight no longer decreased. It was then weighed as the dry weight (D), and the W/D ratio was calculated.

### Detection of total cell count and total protein concentration in bronchoalveolar lavage fluid

The BALF was centrifuged at 4°C for 10 min at a speed of 3,000 rpm/min, and the cell precipitate and supernatant were separated. A cell-counting plate was used to determine the total number of cells in the BALF precipitate. In addition, the total protein concentration in the BALF supernatant was determined using the test kits.

### Hematoxylin and eosin and masson staining of lung tissue

After 24 h of model generation and drug administration, the mice were euthanized, and the lung tissues from each group were collected, fixed in formalin solution, embedded in paraffin, divided into 3 μm sections, routinely stained with HE, and sealed with neutral balsam. Histopathological changes in each group were observed under a light microscope. The inflammation score of the HE staining was evaluated based on a previous study (Cui J. et al., 2020; Supplementary Table S1). In addition, the lung tissue sections were stained with Masson’s trichrome to evaluate collagen deposition in the lungs; collagen deposition was quantified using ImageJ, and the Masson staining score was calculated based on a previous study (Zhou et al., 2016, Supplementary Table S2)

### Enzyme-linked immunosorbent assay

The level of inflammatory factors IL-6, IL-1β, TNF-α, IL-4, and IL-10 in the supernatant of the BALF and serum were determined using ELISA kit, according to the manufacturer’s instructions. Lung tissue (100 mg) was added to 900 μl of saline, homogenized with an ultrasonicator, and centrifuged at 3,000 rpm/min for 15 min; the supernatant was then collected, and the protein levels in the tissue homogenate were homogenized using a total protein test kit to detect Arg-1 levels.

### Biochemical indicator test of lung tissue

The total protein test kit was used to homogenize the protein levels and detect the activity of SOD, GSH-Px, iNOS, and MPO, and the level of MDA in the lung tissue homogenates; all procedures were performed according to the manufacturer’s protocol.

### Flow cytometry analysis of macrophage polarization and neutrophil expression

The BALF was collected in individual Falcon flow tubes and labeled with the sample number. Each BALF sample was divided into two equal aliquots and centrifuged at 400 g for 5 min; the supernatant was discarded, and the precipitate was dried. Following cell washing, 1 μl of PE anti-mouse CD86 and APC anti-mouse CD206 antibodies were added to each tube of the first cell precipitation aliquot and labeled as M1 and M2 macrophages, respectively; negative control and blank control groups were also established. To label the neutrophils, 1 μl each of APC anti-mouse Ly6G and PE anti-mouse CD11b antibodies were added to each tube in the second cell precipitation aliquot, along with negative control and blank control groups. The antibodies were gently mixed to ensure complete fusion of the antibodies and cells. The cells were incubated at 4°C for 20–30 min, protected from light, and then washed, and fixed. The samples were studied using a FACScan flow cytometer (BD Biosciences, Franklin Lakes, NJ, United States), and the flow data was analyzed using FlowJo software.

### Metabolomics analysis

Hundred milligrams of lung tissue was weighed out, added to 500 μl of 80% methanol solution, vortexed for 5 min, homogenized with an ultrasonicator, and centrifuged at 15,000 g for 20 min. The supernatant was then collected and diluted with water to achieve a final methanol concentration of 53%; it was then centrifuged at 15,000 g for 20 min. The supernatant was obtained for untargeted metabolomic analysis using liquid chromatography-mass spectrometry (LC-MS). The data processing and analysis performed, as well as the chromatographic and mass spectrometric conditions, were as described in our previously published paper (Xie et al., 2021).

### Statistical processing

Statistical analysis of the data was performed using the SPSS 20.0 statistical software and was expressed as the mean ± standard deviation. A one-way ANOVA (analysis of variance) with Tukey’s HSD (honest significant difference) post-hoc test was used for comparisons between groups. Differences were considered statistically significant at *p* < 0.05.

## Results

### Identification of the main compounds in Jian-Ti-Kang-Yi decoction using UPLC-MS analysis

Astragaloside IV, atractylenolide III, cimicifugoside, 5-O-methylvisammioside, isoimperatorin, harpagide, oleanolic acid, platycodin D, chlorogenic acid, cynaroside, forsythiaside, phillyrin, rosmarinic acid, patchouli alcohol, and liquiritin were used as the reference standards to validate the main compounds in JTKY. Detailed information on these compounds is shown in Supplementary Figure S2. Typical base peak intensity (BPI) chromatograms of JTKY and the reference standards are shown in Supplementary Figure S3. The characteristic fragment ions of these compounds are listed in Supplementary Table S3 (Supplementary Material). Astragaloside IV in *Astragalus mongholicus* Bunge, atractylenolide III in *Atractylodes macrocephala* Koidz., cimicifugoside, 5-O-methylvisammioside in *Saposhnikovia divaricata* (Turcz. ex Ledeb.) Schischk., isoimperatorin in *Glehnia littoralis* (A. Gray) F. Schmidt ex Miq., harpagide in *Ophiopogon japonicus* (Thunb.) Ker Gawl., oleanolic acid in *Ganoderma lucidum* (Curtis) P. Karst, platycodin D in *Platycodon grandiflorus* (Jacq.) A. DC., chlorogenic acid and cynaroside in *Lonicera japonica* Thunb., forsythiaside and phillyrin in *Forsythia suspensa* (Thunb.) Vahl, rosmarinic acid in *Perilla frutescens* (L.) Britton, patchouli alcohol in *Agastache rugosa* (Fisch. & C. A. Mey.) Kuntze, and liquiritin in *Glycyrrhiza uralensis* Fisch. ex DC. were identified as the predominant compounds in JTKY.

### Therapeutic effects of Jian-Ti-Kang-Yi decoction on poly(I:C)-induced pneumonia model mice

**FIGURE 1:**
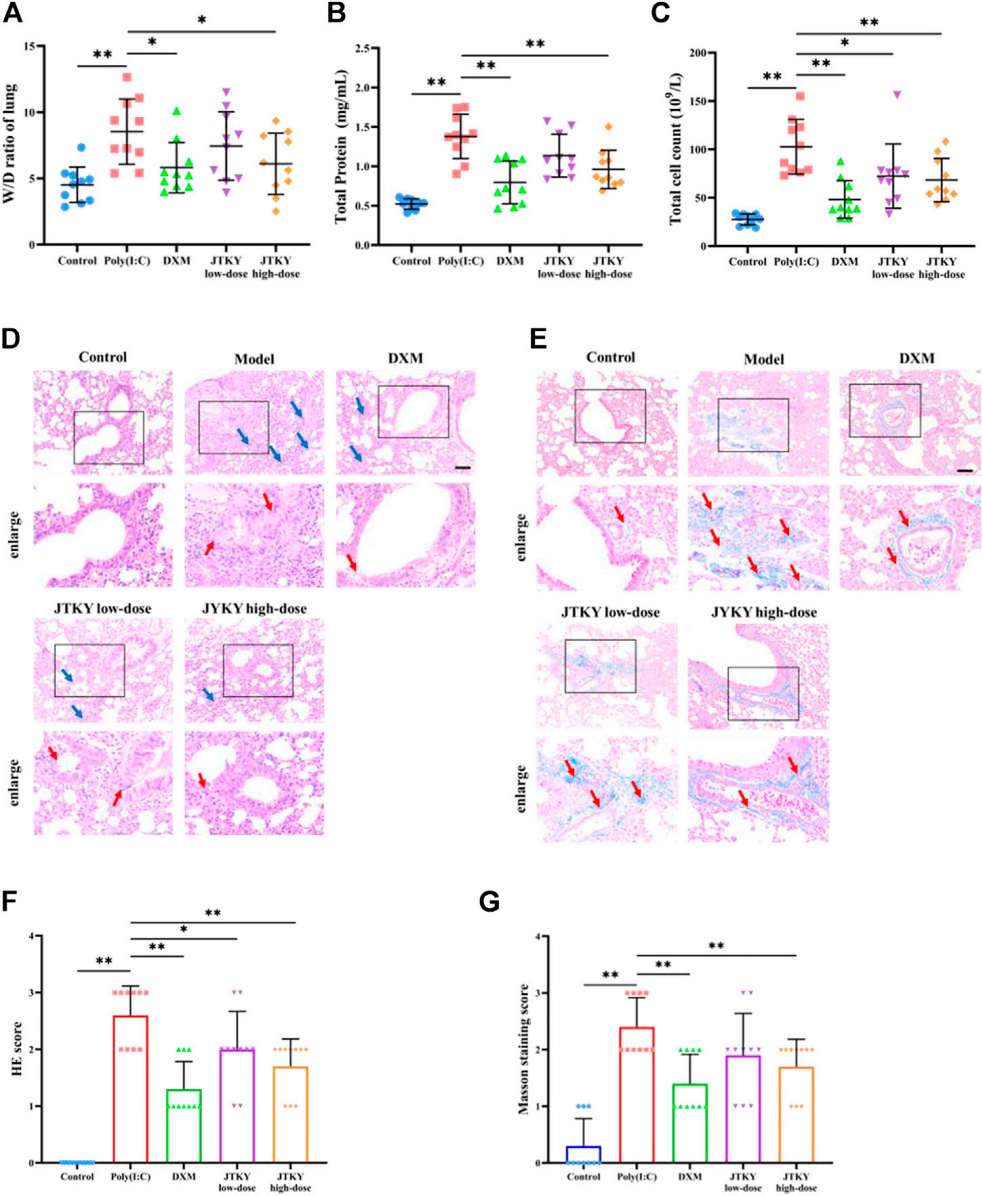
JTKY ameliorated poly(I:C)-induced viral pneumonia. **(A)** JTKY treatment decreased the W/D ratio in lung tissue. **(B)** JTKY treatment decreased the total protein concentration in BALF. **(C)** JTKY treatment decreased the total cell count in BALF. **(D, F)** HE staining indicated that JTKY treatment alleviated the pathological changes and decreased the inflammation score in lung (100 and ×200), blue arrows indicated the infiltration of inflammatory cells and red arrows indicated the damage of bronchial epithelial structure. **(E, G)** Masson staining showed that JTKY treatment decreased the collagen contents in lung (100 and ×200), red arrows indicated the accumulation of collagen contents. Control, Poly(I:C), DXM, JTKY low-dose and JTKY high-dose groups (*n* = 10 per group). *: *p* < 0.05; **: *p* < 0.01.

The W/D ratio of lung tissue, total protein level, and total cell count in the BALF were higher in the poly(I:C) group than in the control group (*p* < 0.01). The W/D ratio (*p* < 0.05, Figure 1A) and total protein level in the BALF (*p* < 0.01, Figure 1B) were lower in the DXM and JTKY high-dose groups, compared to those in the poly(I:C) group. The total cell count in the BALF was higher in the poly(I:C) group than in the DXM, JTKY low-dose, and JTKY high-dose groups (*p* < 0.01, *p* < 0.05, and *p* < 0.01, respectively; Figure 1C).

HE staining of the lung tissue in the control group showed an intact bronchial epithelial structure, normal interalveolar septum, and the absence of interstitial edema and significant inflammatory cell exudates. In the poly(I:C) group, the bronchial epithelial structure was no longer intact and infiltration of a considerable number of inflammatory cells was observed. Treatment with DXM and low- and high-doses of JTKY significantly improved the pathological changes in the lung tissue of poly(I:C)-induced pneumonia model mice (Figure 1D). The inflammation score of HE staining was also higher in the poly(I:C) group than in the control group, and lower in DXM, JTKY low-dose, and JTKY high-dose groups compared to that in the poly(I:C) group (*p* < 0.01, *p* < 0.05, and *p* < 0.01, respectively; Figure 1F). Masson staining showed that collagen deposition was increased in the poly(I:C) group compared to that in the control group (*p* < 0.01), whereas DXM and high-dose JTKY treatment decreased the deposition of collagen in the lungs of poly(I:C)-induced pneumonia model mice (*p* < 0.01, Figures 1E,G).

### Anti-inflammatory effects of Jian-Ti-Kang-Yi decoction on poly(I:C)-induced pneumonia model mice

**FIGURE 2:**
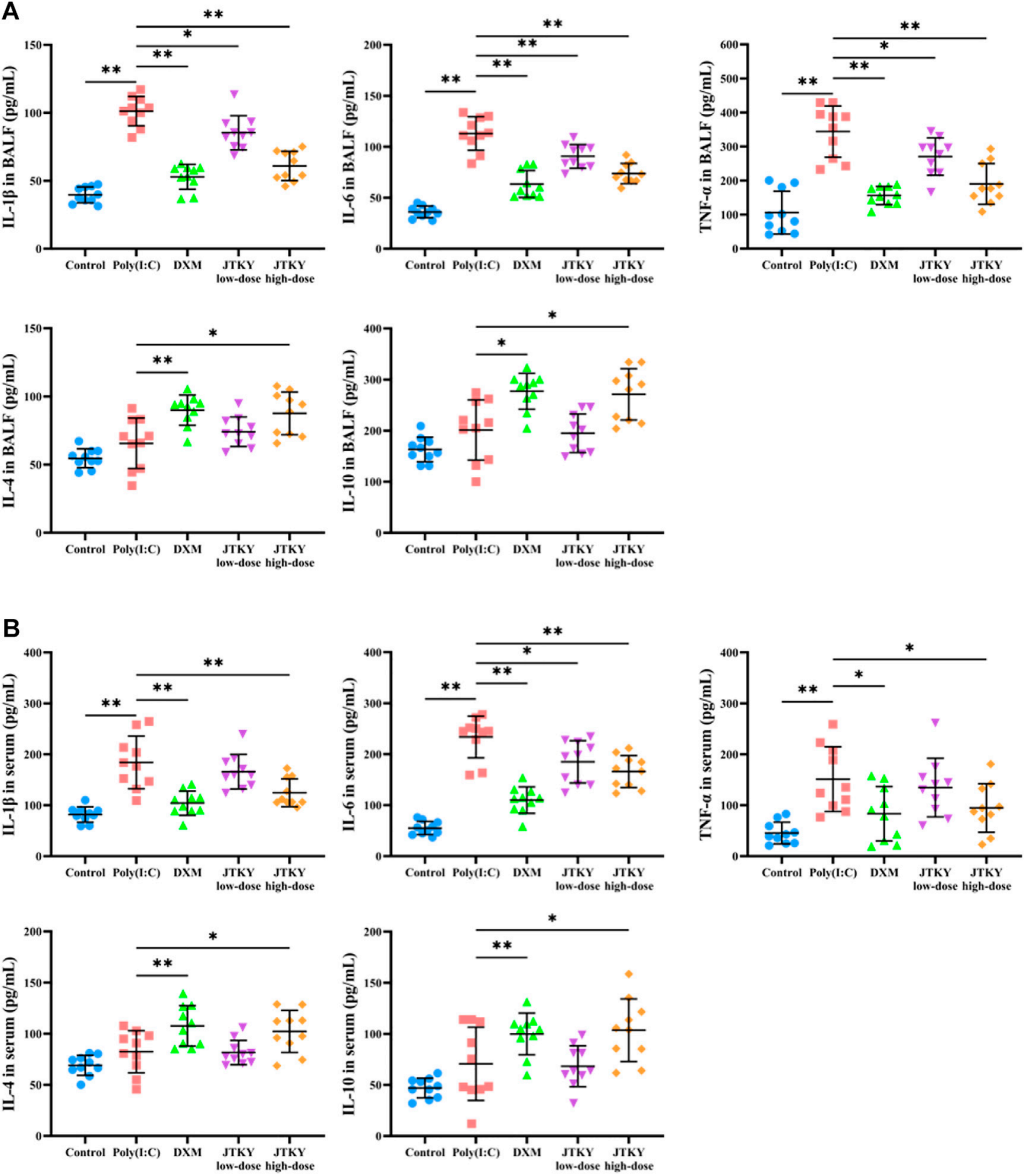
JTKY affected the levels of cytokines in poly(I:C)-induced viral pneumonia model mice. **(A,B)** JTKY treatment decreased the levels of IL-1β, IL-6 and TNF-αand increased the levels of IL-4 and IL-10 in BALF **(A)** and serum **(B)**. Control, poly(I:C), DXM, JTKY low-dose and JTKY high-dose groups (*n* = 10 per group). *: *p* < 0.05; **: *p* < 0.01.

We used ELISA kits to measure the levels of pro-inflammatory cytokines (IL-6, IL-1β, and TNF-α) and cytokines with immunomodulatory effects (IL-4 and IL-10) in the BALF and serum. The levels of IL-1β, IL-6, and TNF-α in the BALF were significantly increased in the poly(I:C) group (*p* < 0.01) compared to those in the control group. The levels of IL-1β (*p* < 0.01, *p* < 0.05, and *p* < 0.01, respectively), IL-6 (*p* < 0.01), and TNF-α (*p* < 0.01, *p* < 0.05, and *p* < 0.01, respectively) in the BALF were significantly decreased in the DXM, JTKY low-dose, and JTKY high-dose groups, compared to those in the poly(I:C) group. There were no significant differences in the IL-4 and IL-10 levels between the control and poly(I:C) groups. However, the levels of IL-4 (*p* < 0.01 and *p* < 0.05, respectively) and IL-10 (*p* < 0.01 and *p* < 0.05, respectively) in the BALF were increased in the DXM and JTKY high-dose groups, compared to those in the poly(I:C) group (Figure 2A).

Moreover, the serum levels of IL-1β, IL-6, and TNF-α were significantly increased in the poly(I:C) group (*p* < 0.01) compared to those in the control group, and the serum levels of IL-1β (*p* < 0.01) and TNF-α (*p* < 0.05) were significantly decreased in the DXM and JTKY high-dose groups. The IL-6 level (*p* < 0.01, *p* < 0.05, and *p* < 0.01, respectively) in the serum was significantly lower in the DXM, JTKY low-dose, and JTKY high-dose groups compared to that in the poly(I:C) group. No significant differences were observed in the serum levels of IL-4 and IL-10 between the control and poly(I:C) groups. However, DXM and high-dose JTKY treatment increased the levels of IL-4 (*p* < 0.01 and *p* < 0.05, respectively) and IL-10 (*p* < 0.01 and *p* < 0.05, respectively) in the serum of poly(I:C)-induced pneumonia mice (Figure 2B).

**FIGURE 3:**
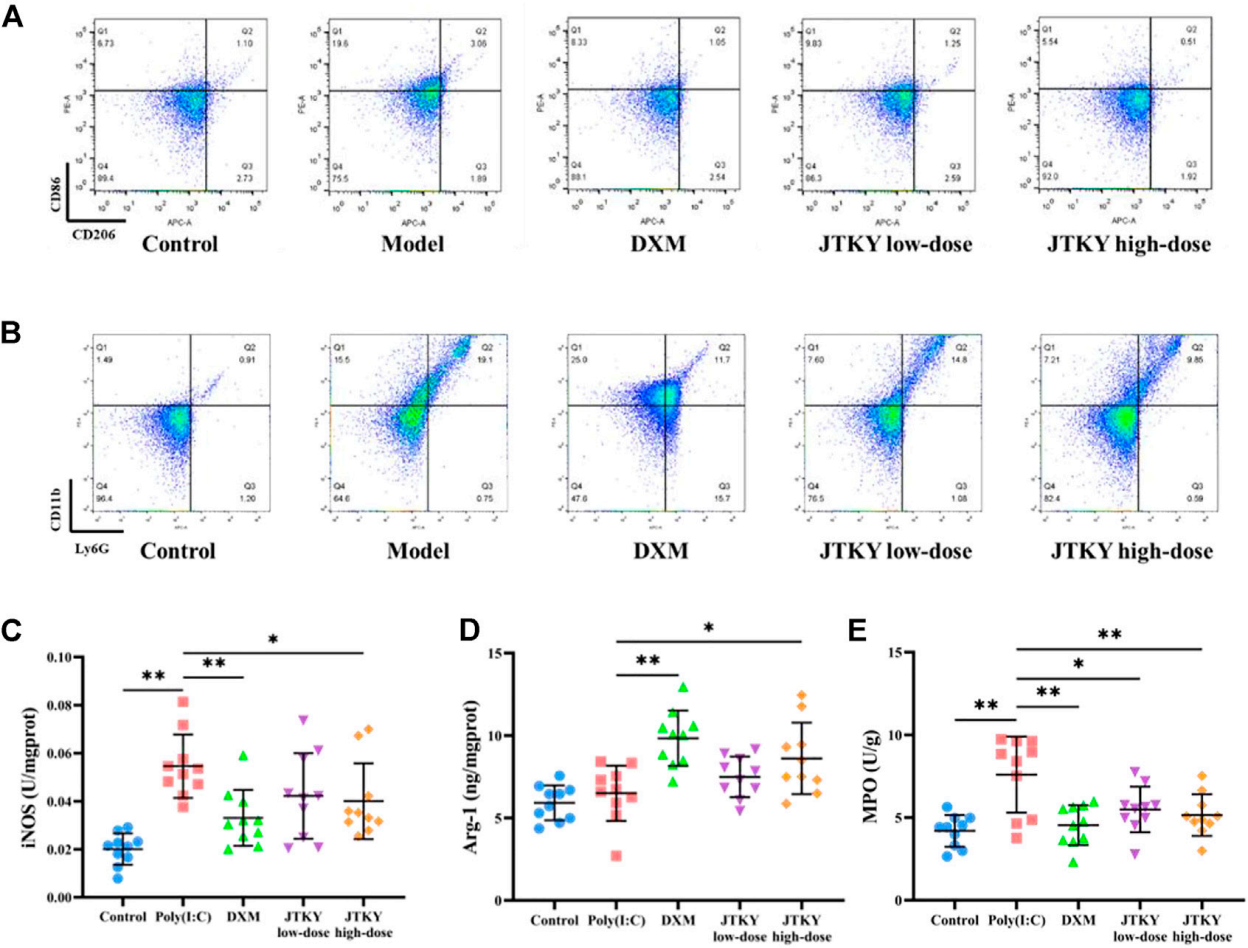
JTKY treatment affected the polarization of macrophage and inhibited the infiltration of neutrophils in lung. **(A)** Flow cytometry showed that JTKY treatment decreased the ratio between CD86^+^ and CD206^+^ cells **(A)** and the proportion of Ly6G^+^CD11b^+^ cells **(B)** in BALF. **(C,D)** JTKY treatment decreased the activity of iNOS **(C)** and increased the level of Arg-1 **(D)** in lung tissue homogenates. **(E)** JTKY treatment decreased the activity of MPO in lung tissue homogenates. Control, poly(I:C), DXM, JTKY low-dose and JTKY high-dose groups (*n* = 10 per group). *: *p* < 0.05; **: *p* < 0.01.

In addition, we measured the proportion of CD86^+^ (cell marker of M1 macrophages) and CD206^+^ (cell marker of M2 macrophages) cells, the activity of iNOS (mainly produced by M1 macrophages), and the level of Arg-1 (mainly produced by M2 macrophages) in lung tissue homogenates. The ratio of CD86^+^/CD206^+^ cells in the BALF in poly(I:C) group was higher than in the control group (*p* < 0.01). The ratio of CD86^+^/CD206^+^ cells in the BALF was lower in the DXM, JTKY low-dose, and JTKY high-dose groups (*p* < 0.01, Figure 3A; Table 2) compared to that in the poly(I:C) group. In addition, the activity of iNOS was higher in the poly(I:C) group than in the control group (*p* < 0.01). DXM and high-dose JTKY treatment decreased the activity of iNOS (*p* < 0.01 and *p* < 0.05, respectively; Figure 3C) and increased the level of Arg-1 (*p* < 0.01 and *p* < 0.05, respectively; Figure 3D) in lung tissue homogenates.

**TABLE 2:** Effects of JTKY on the proportions of CD86^+^ and CD206^+^ cells in BALF.

Group	CD86^+^ (%)	CD206^+^ (%)	CD86^+^/CD206^+^
Control	6.60 ± 0.67	2.61 ± 0.46	2.67 ± 0.77
Poly(I:C)	20.41 ± 0.68^##^	1.64 ± 0.18	12.59 ± 1.58^##^
DXM	11.51 ± 2.37**	2.94 ± 0.32	3.86 ± 0.42**
JTKY low-dose	11.44 ± 1.39**	2.51 ± 0.17**	4.62 ± 0.87**
JTKY high-dose	7.68 ± 1.74**	2.50 ± 0.77	3.17 ± 0.50**

Control, poly(I:C), DXM, JTKY low-dose, and JTKY, high-dose groups (*n* = 10 per group). ^##^: *p* < 0.01 compared with the control group; *: *p* < 0.05 compared with the poly(I:C) group; **: *p* < 0.01 compared with the poly(I:C) group.

We also investigated the proportion of Ly6G^+^CD11b^+^ (neutrophils) cells in the BALF and the activity of MPO (an important factor in neutrophils) in the lung tissue homogenates. The proportion of Ly6G^+^CD11b^+^ cells increased, and the activity of MPO was higher in the poly(I:C) group than in the control group (*p* < 0.01). DXM, low-dose JTYK, and high-dose JTKY treatment decreased the proportion of Ly6G^+^CD11b^+^ cells in the BALF (*p* < 0.01, Figure 3B; Table 3); the activity of MPO was also lower in these groups compared to that in the poly(I:C) group (*p* < 0.01, *p* < 0.05, and *p* < 0.01, respectively; Figure 3E).

**TABLE 3:** Effects of JTKY on the proportion of Ly6G^+^CD11b^+^ cells in BALF.

Group	Ly6G^+^CD11b^+^ (%)
Control	1.31 ± 0.28
Poly(I:C)	19.49 ± 1.24^##^
DXM	10.04 ± 1.21**
JTKY low-dose	12.46 ± 1.66**
JTKY high-dose	9.81 ± 2.13**

Control, poly(I:C), DXM, JTKY low-dose, and JTKY, high-dose groups (*n* = 10 per group). ^##^: *p* < 0.01 compared with the control group; **: *p* < 0.01 compared with the poly(I:C) group.

### Anti-oxidative effects of Jian-Ti-Kang-Yi decoction on poly(I:C)-induced pneumonia model mice

The activities of SOD and GSH-Px, and the level of MDA in lung tissue homogenates were measured to evaluate the anti-oxidative effects of JTKY. SOD and GSH-Px activities were lower and MDA level was higher in the poly(I:C) group (*p* < 0.01) compared to those in the control group. The activities of SOD (*p* < 0.01, *p* < 0.05, and *p* < 0.01, respectively) and GSH-Px (*p* < 0.01, *p* < 0.05, and *p* < 0.01, respectively) increased and the levels of MDA (*p* < 0.01, *p* < 0.05, and *p* < 0.01, respectively) decreased in the DXM, JTKY low-dose, and JTKY high-dose groups compared to those in the poly(I:C) group (Table 4).

**TABLE 4:** Effects of JTKY on the activities of SOD and GSH-Px and the level of MDA in lung tissue homogenates.

Group	SOD (U/mgprot)	MDA (nmol/mgprot)	GSH-Px (U/mgprot)
Control	53.37 ± 8.38	1.74 ± 0.31	22.49 ± 4.07
Poly(I:C)	24.48 ± 6.97^##^	3.76 ± 1.19^##^	10.10 ± 3.61^##^
DXM	39.83 ± 7.31**	2.31 ± 0.53**	15.99 ± 5.07**
JTKY low-dose	32.73 ± 8.03*	2.87 ± 0.51*	14.13 ± 4.23*
JTKY high-dose	37.35 ± 4.64**	2.56 ± 0.51**	15.81 ± 3.4**

Control, poly(I:C), DXM, JTKY low-dose, and JTKY, high-dose groups (*n* = 10 per group). ^##^: *p* < 0.01 compared with the control group; *: *p* < 0.05 compared with the poly(I:C) group; **: *p* < 0.01 compared with the poly(I:C) group.

Thus, a high dose of JTKY could significantly improve pathological changes in the lung tissue, inhibit inflammatory responses, and alleviate oxidative stress in poly(I:C)-induced pneumonia. Therefore, a high dose of JTKY was selected for subsequent studies on the effects of JTKY on the metabolites in poly(I:C)-induced pneumonia.

### Modulatory effects of Jian-Ti-Kang-Yi decoction on metabolites in the lungs of poly(I:C)-induced pneumonia model mice

**FIGURE 4:**
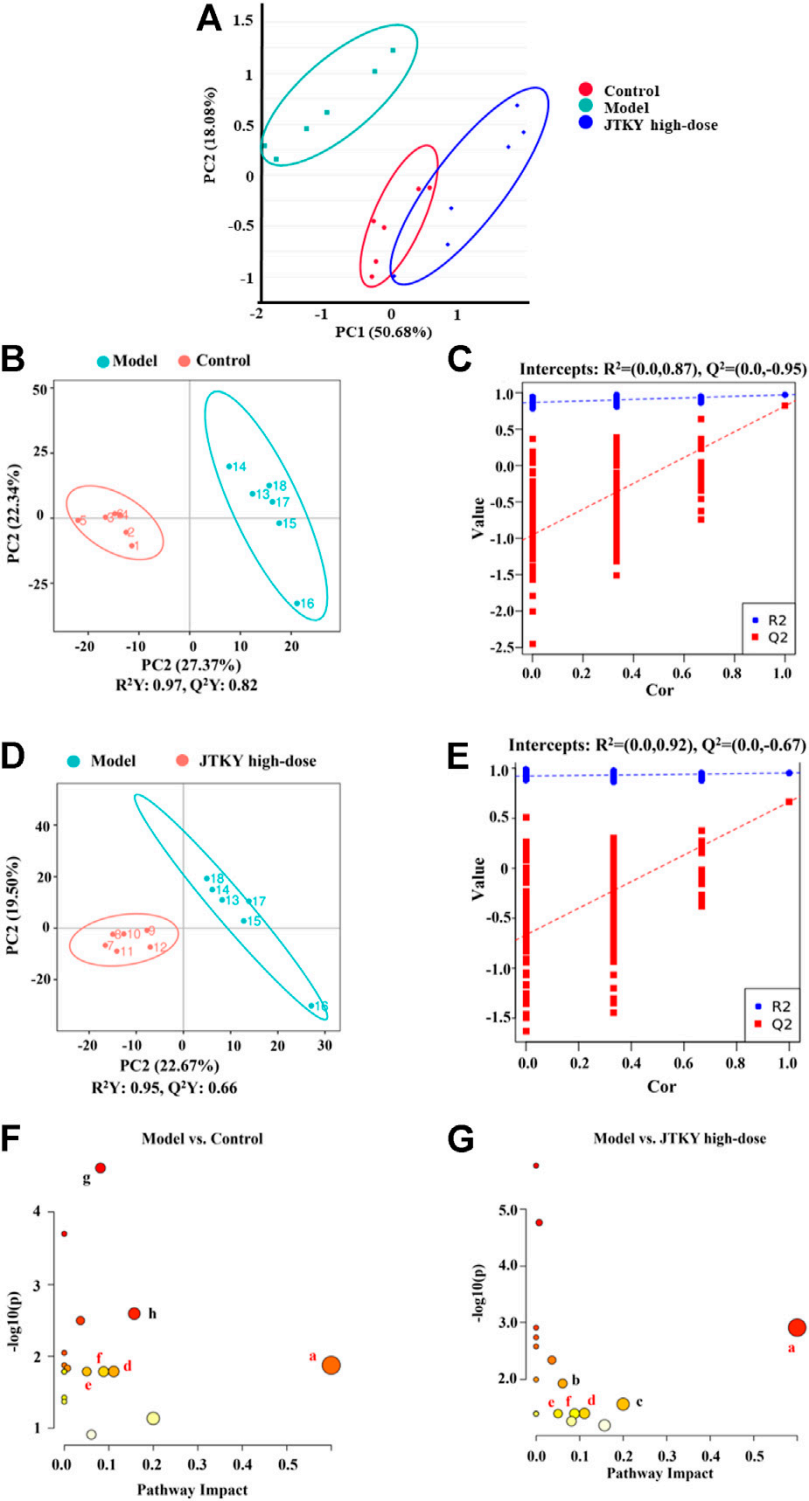
JTKY treatment modulated the metabolites in lung in poly(I:C)-induced viral pneumonia model mice. **(A)** Scores plots of PCA among each group. **(B,C)** Scores plots of PLS-DA between the control and poly(I:C) groups and the corresponding coefficient of loading plots. **(D,E)** Scores plots of PLS-DA between the model and JTKY high-dose groups and the corresponding coefficient of loading plots. **(F,G)** Summary of pathway analysis between control and poly(I:C) groups **(F)** and between model and JTKY high-dose groups **(G)**, the sames pathways were written in red. a Synthesis and degradation of ketone bodies; b Arginine and proline metabolism; c Biotin metabolism; d Butanoate metabolism; e Alanine, aspartate and glutamate metabolism; f Citrate cycle (TCA cycle); g Amino sugar and nucleotide sugar metabolism; h Fructose and mannose metabolism. Control, Poly(I:C), and JTKY high-dose groups (*n* = 6 per group).

Several studies have demonstrated a close relationship between metabolism and virus-induced acute lung injury (Nolan et al., 2021). Modulation of metabolism could provide a new approach for the treatment of lung injury (Zhong et al., 2018). An oral solution of Pu-Di-Lan has been shown to alleviate acute lung injury by regulating the levels of aspartate and L-cysteine and inhibiting the activation of the NF-ĸB pathway (Tian et al., 2020). *Lonicera japonica* Thunb. and *Forsythia suspensa* (Thunb.) Vahl have been shown to inhibit inflammatory responses in a mouse model of H1N1-induced pneumonia and simultaneously regulate galactose, glycine, serine, and threonine metabolism, as well as the synthesis and degradation of ketone bodies (Qian et al., 2018). Therefore, untargeted metabolomics were used to elucidate the metabolic modulatory effects of JTKY on poly(I:C)-induced pneumonia model mice. The principal component analysis (PCA) plot showed that the control and poly(I:C) groups as well as the model and JTKY high-dose groups were both well differentiated (Figure 4A). To identify the differentially expressed metabolites, a partial least squares-discriminant analysis (PLS-DA) model was used, and the explanatory power (R^2^) and predictive power (Q^2^) of the constructed PLS-DA model were assessed. R^2^ = 0.87 and Q^2^ = −0.95 for the poly(I:C) group compared to the control group, whereas R^2^ = 0.92 and Q^2^ = −0.67 for the JTKY high-dose group compared to the poly(I:C) group (Figures 4B–E). These results show that the model was stable and had good predictive power.

The following two criteria were used to screen for differentially expressed metabolites: *p* < 0.05 and VIP > 1.0; 26 differentially expressed metabolites were identified (Table 5). The levels of nonanoic acid, xanthurenic acid, arachidic acid, fumaric acid, acetoacetate, pyroglutamic acid, L-homocitrulline, and prostaglandin A2 were significantly increased, whereas the levels of d-glucosamine, l-adrenaline, l-fucose, palmitoleic acid, oleanolic acid, alpha-ketoglutaric acid, L-asparagine, D-mannose 6-phosphate, L-pipecolate, gentisic acid, ornithine, 7-alpha-hydroxytestosterone, D-beta-hydroxybutyric acid, and docosapentaenoic acid were significantly decreased in the poly(I:C) group compared to those in the control group. Levels of alpha-ketoglutaric acid, biotin, fumaric acid, L-asparagine, acetoacetate, L-pipecolate, ornithine, and D-beta-hydroxybutyric acid were significantly increased in the JTKY high-dose group, whereas the levels of hydroxyproline, nonanoic acid, kynurenic acid, pyroglutamic acid, L-homocitrulline, xanthurenic acid, deoxycholic acid, prostaglandin A2, arachidic acid, and docosapentaenoic acid were significantly decreased in the JTKY high-dose group compared to those in the poly(I:C) group.

**TABLE 5:** Differential metabolites in poly(I:C)-induced viral pneumonia model mice after the treatment of JTKY.

Formula	RT (min)	m/z	Metabolites	VIP		FC		Trend		Pathway
				P vs. C	J vs. M	P vs. C	J vs. P	P vs. C	J vs. P	
C_5_H_6_O_5_	1.48	145.01	alpha-Ketoglutaric acid	1.29	1.54	0.63	1.49	↓^#^	↑*	d, e, f
C_10_H_16_N_2_O_3_S	5.35	245.10	Biotin	1.64	1.53	0.70	1.45	↓	↑*	c
C_4_H_4_O_4_	2.39	115.00	Fumaric acid	1.71	1.49	0.67	1.93	↓^#^	↑*	e, f
C_4_H_8_N_2_O_3_	1.33	133.06	L-Asparagine	1.29	1.85	0.71	1.47	↓^#^	↑**	e
C_4_H_6_O_3_	1.48	101.02	Acetoacetate	1.14	2.05	0.70	1.56	↓^#^	↑**	a, d
C_6_H_13_O_9_P	1.53	259.02	D-Mannose 6-phosphate	1.26	1.99	0.67	1.30	↓^#^	↑	g, h
C_6_H_13_NO_5_	1.43	180.09	D-Glucosamine	1.93	1.50	0.48	1.25	↓^##^	↑	g
C_6_H_11_NO_2_	1.15	130.09	L-Pipecolate	1.28	2.09	0.62	1.65	↓^#^	↑*	
C_7_H_6_O_4_	5.64	153.02	Gentisic acid	1.55	1.70	0.45	1.27	↓^#^	↑	
C_9_H_13_NO_3_	5.56	184.10	L-Adrenaline	1.01	2.12	0.47	1.96	↓^##^	↑**	
C_6_H_12_O_5_	1.41	199.04	L-Fucose	1.66	1.14	0.42	1.49	↓^##^	↑	g, h
C_5_H_9_NO_3_	1.35	132.07	Hydroxyproline	1.90	1.56	1.36	0.50	↑	↓*	b
C_9_H_18_O_2_	5.94	157.12	Non-anoic acid	1.24	1.64	1.45	0.51	↑^##^	↓**	
C_5_H_12_N_2_O_2_	1.23	133.10	Ornithine	1.93	1.77	0.30	1.43	↓^#^	↑**	
C_10_H_7_NO_3_	5.38	190.05	Kynurenic acid	2.17	1.26	1.38	0.46	↑	↓**	
C_5_H_7_NO_3_	6.39	130.05	Pyroglutamic acid	1.40	1.79	1.58	0.16	↑^#^	↓**	
C_7_H_15_N_3_O_3_	1.45	190.12	L-Homocitrulline	1.15	1.41	1.43	0.58	↑^#^	↓**	
C_10_H_7_NO_4_	5.30	206.04	Xanthurenic acid	1.52	1.60	1.53	0.61	↑^##^	↓**	
C_24_H_40_O_4_	6.94	391.29	Deoxycholic acid	1.13	1.32	1.45	0.19	↑	↓*	
C_19_H_28_O_3_	6.02	305.21	7α-Hydroxytestosterone	1.24	1.28	0.56	0.82	↓^#^	↓	
C_4_H_8_O_3_	2.99	103.04	D-beta-Hydroxybutyric acid	1.23	2.11	0.69	1.74	↓^#^	↑*	a
C_20_H_30_O_4_	7.37	333.21	Prostaglandin A2	2.07	1.31	1.48	0.22	↑^#^	↓**	
C_20_H_40_O_2_	11.56	311.30	Arachidic acid	1.86	1.15	1.30	0.33	↑^##^	↓**	
C_16_H_30_O_2_	7.85	255.23	Palmitoleic acid	1.41	1.66	0.66	0.85	↓^##^	↓	
C_22_H_34_O_2_	10.19	331.26	Docosapentaenoic acid	1.20	1.93	0.61	0.74	↓^#^	↓*	
C_30_H_48_O_3_	8.33	439.36	Oleanolic acid	1.72	1.24	0.33	1.25	↓^##^	↑	

Control, poly(I:C), and JTKY high-dose groups (*n* = 6 per group). RT, retention time; VIP, variable importance of projection; FC, fold change; ^#^: *p* < 0.05 as compared to the control group; ^##^: *p* < 0.01 as compared to the control group; *: *p* < 0.05 as compared to the poly(I:C) group; **: *p* < 0.01 as compared to the poly(I:C) group; ↑, content increased; ↓, content decreased; vs., versus; C, control group; P, poly(I:C) group; J, JTKY high-dose group a, Synthesis and degradation of ketone bodies; b, Arginine and proline metabolism; c, Biotin metabolism; d, Butanoate metabolism; e, Alanine, aspartate and glutamate metabolism; f, TCA, cycle; g, Amino sugar and nucleotide sugar metabolism; h, Fructose and mannose metabolism.

### Pathway analysis of differential metabolites in poly(I:C)-induced pneumonia model mice following Jian-Ti-Kang-Yi decoction treatment

The MetaboAnalyst platform (https://www.metaboanalyst.ca/) was used to screen the related metabolic pathways between the control and poly(I:C) groups, and between the model and JTKY high-dose groups. Differential metabolic pathways were selected based on a pathway impact of > 0.05 and *p* < 0.05. Differential metabolic pathways between the control and poly(I:C) groups included the synthesis and degradation of ketone bodies, and the metabolism of butanoate, alanine, aspartate, glutamate, the tricarboxylic acid cycle (TCA cycle), amino sugar, nucleotide sugar, fructose, and mannose (Figure 4F). Differential metabolic pathways between the poly(I:C) and the JTKY high-dose groups included the synthesis and degradation of ketone bodies, and the metabolism of arginine, proline, biotin, butanoate, alanine, aspartate, glutamate, and the TCA cycle (Figure 4G). Amongst these pathways, the synthesis and degradation of ketone bodies, and the butanoate metabolism, alanine, aspartate, and glutamate metabolism, and the TCA cycle were common to the control, model, and JTKY high-dose groups.

## Discussion

A mouse model of pneumonia was generated in this study through the intratracheal injection of poly(I: C). Poly(I:C) is a synthetic double-stranded ribonucleic acid that is frequently used in viral pneumonia models because it mimics the immune activation mechanism of viral infections and is a component of the pathological process of cytokine storms in viral pneumonia (Maelfait et al., 2008; Bao et al., 2022). Our findings indicate that the W/D ratio of the lung tissue in the mice was increased in the poly(I:C) group, while the total cell count and protein levels in the BALF were also significantly increased, implying increased lung tissue permeability. Pathological staining revealed significant infiltration of inflammatory cells into the lung tissue of the mice in the poly(I:C) group, with bronchial epithelial cell damage. These changes were consistent with viral pneumonia, implying that the model was successfully established. JTKY significantly decreased lung tissue permeability in the poly(I:C)-induced pneumonia model mice, and improved the histopathological changes in their lungs. Furthermore, DXM was used as the positive control; DXM is a commonly used anti-inflammatory drug in clinical practice, and can be used as a positive control in studies using a poly(I:C)-induced pneumonia model (Cui J. et al., 2020). Our findings indicated that there was no significant difference between the high-dose JTKY group and the positive drug group in improving lung tissue permeability and pathological changes, implying that JTKY has a significant therapeutic effect on viral pneumonia.

Next, we investigated the effects of JTKY on the inflammatory response in poly(I:C)-induced pneumonia model mice. The results indicated that JTKY decreased IL-6, IL-1β, and TNF-α levels in the BALF while increasing IL-4 and IL-10 levels. Pro-inflammatory cytokines, such as IL-6, IL-1β, and TNF-α, are significantly increased during viral infections and exacerbate lung tissue damage. IL-4 and IL-10 are immunomodulatory cytokines that inhibit the development of inflammatory responses in the lungs (Huang et al., 2019). Macrophages play a critical role in the inflammatory response during viral pneumonia. Flow cytometry analysis revealed that JTKY decreased the proportion of M1 macrophages in the BALF while increasing the proportion of M2 macrophages. Additionally, JTKY decreased iNOS levels in the lung tissue and increased Arg-1 levels. After stimulation, macrophages can be classified as either M1-type, with pro-inflammatory effects, or M2-type, with anti-inflammatory effects (Yunna et al., 2020). Numerous studies have demonstrated that acute viral infections can induce M1 macrophage polarization, which results in the release of pro-inflammatory factors, such as IL-1β, IL-6, and iNOS (Nejatifard et al., 2021). These factors stimulate the production of additional M1 macrophages and also activate other immune cells, which migrate into the lung tissue. M2 macrophages produce immunomodulatory cytokines such as IL-4, IL-10, and Arg-1. The proportion of M2 macrophages is lower during viral infections (Laskin et al., 2018). Inhibition of the polarization of M1 macrophages and promotion of the polarization of M2 macrophages can alleviate the inflammatory response associated with viral pneumonia. Additionally, the massive activation of neutrophils contributes significantly to cytokine storms. The results indicated that JTKY treatment decreased the neutrophil ratio and MPO levels in the lungs of poly(I:C)-induced pneumonia model mice. Neutrophils contribute significantly to innate immunity through the phagocytosis of microbial pathogens or by limiting pathogen invasion *via* the formation of neutrophil extracellular traps (NETs) (Amulic et al., 2012). When a pathogen infects the body, it can cause excessive neutrophil activation and the release of NETs, which can induce the polarization of M1-type macrophages, resulting in the development of a cytokine storm and aggravating acute lung injury (Song et al., 2019). Additionally, MPO is a significant component of cytoplasmic granules, and its level directly correlates with neutrophil activation and infiltration. Excessive neutrophil activation generates large amounts of MPO, resulting in an inflammatory response and lung tissue damage (Aratani, 2018).

Oxidative stress is also a significant pathological response in viral pneumonia; a viral respiratory tract infection directly results in the production of excessive reactive oxygen species (ROS) in lung tissue. Meanwhile, high levels of inflammatory factors also promote the production of ROS and damage lung tissue. Our findings indicated that JTKY decreased MDA levels and increased SOD and GSH-Px activities in the lung tissue of poly(I:C)-induced pneumonia model mice. MDA is a cytotoxic end product of lipid peroxidation and its level is associated with oxidative stress (Tsikas, 2017). SOD and GSH-Px are antioxidant enzymes that participate in ROS scavenging (Olsvik et al., 2005). SOD promotes the conversion of ROS to H_2_O_2_, which is then catalyzed by GSH-Px to form H_2_O and O_2_. Increasing the activities of SOD and GSH-Px protects the lung tissue from oxidative stress-induced injury (Liu Z. et al., 2022).

Moreover, we used untargeted metabolomics to examine the effect of JTKY on the metabolites found in the lung tissue of poly(I:C)-induced pneumonia model mice. PCA and PLS-DA revealed that the metabolism of the lung tissue in poly(I:C)-induced pneumonia model mice treated with JTYK was significantly altered. Additional differential metabolite analysis revealed that JTKY might affect the levels of 26 metabolites, including L-asparagine, L-adrenaline, ornithine, and alpha-ketoglutaric acid. Metabolic pathway analysis of the differential metabolites using MetaboAnalyst revealed that the synthesis and degradation of ketone bodies, and the metabolism of the TCA cycle, alanine, aspartate, glutamate, and butanoate were the common pathways where changes occurred between the control and poly(I:C) groups and between poly(I:C) and JTKY high-dose groups, thus implying that the effect of JTKY on the treatment of poly(I:C)-induced pneumonia may act *via* the aforementioned pathways.

For synthesis and degradation of ketone bodies, JTKY increased the levels of acetoacetate and D-beta-hydroxybutyric acid in the lungs of mice with viral pneumonia. Acetoacetate, beta-hydroxybutyric acid, and acetone are the intermediate products of hepatic fatty acid oxidative catabolism. According to a study, ketone bodies are critical in regulating the inflammatory response and oxidative stress of the body (Lu et al., 2020). The most abundant component of ketone bodies is D-beta-hydroxybutyric acid, a product of acetoacetate, which inhibits the inflammatory response (Stubbs et al., 2020). D-beta-hydroxybutyric acid inhibits the activation of NLRP3 inflammasomes in macrophages and neutrophils, thereby inhibiting the development of inflammatory responses (Goldberg et al., 2017). Furthermore, a study discovered that D-beta-hydroxybutyric acid can reduce inflammation by inhibiting the activation of the NF-κB pathway by binding to the hydroxycarboxylic acid receptor 2 (HCAR2), a receptor found on the surface of macrophages (Rahman et al., 2014). Cells can also oxidize D-beta-hydroxybutyric acid, resulting in the production of NADPH in the cytoplasm, which protects the cells from oxidative stress injury (Haces et al., 2008). The mechanism by which JTKY inhibits macrophage activation may be associated with the increased production of D-beta-hydroxybutyric acid.

Regarding of TCA cycle, our findings indicate that JTKY increases alpha-ketoglutaric acid and fumaric acid levels in poly(I:C)-induced pneumonia model mice. The TCA cycle is the body’s most efficient way to oxidize sugar for energy and serves as a hub for sugar, lipid, and amino acid metabolism to interact and convert. Recent research has revealed a strong correlation between glucose metabolism reprogramming and macrophage polarization (Russo et al., 2021). Numerous metabolites of the TCA cycle regulate macrophage polarization. By inhibiting the activation of the NF-κB pathway, alpha-ketoglutaric acid inhibits M1 macrophage polarization, decreases the production of the pro-inflammatory factors IL-1β, IL-6, and TNF-α, and promotes M2 macrophage polarization *via* JMJD3 activation (Liu P. S. et al., 2017; Liu S. et al., 2021). Melatonin inhibits the inflammatory response by increasing the level of alpha-ketoglutaric acid in macrophages (Liu Z. et al., 2017). Fumaric acid inhibits mononuclear phagocyte activation by inhibiting the NLRP3 inflammasome (Miglio et al., 2015). Additionally, fumaric acid has been shown to have antioxidant properties *via* the activation of the Nrf2 pathway (Linker et al., 2011). Therefore, the mechanism by which JTKY reduces oxidative stress and inflammation may be related to the regulation of the TCA cycle.

For alanine, aspartate, and glutamate metabolism, our findings indicated that JTKY increased the levels of alpha-ketoglutaric acid, L-asparagine, and fumaric acid in poly(I:C)-induced pneumonia model mice. Additionally, alpha-ketoglutaric acid and fumaric acid are important metabolites of the TCA cycle that act as anti-inflammatory agents (Bailey et al., 2019). Metabolomic analysis has revealed that patients with chronic obstructive pulmonary disease and pneumonia have lower serum levels of L-asparagine (Inoue and Ikeda, 2019). However, the precise relationship between L-asparagine and viral pneumonia remains unknown.

As the results of butanoate metabolism, our findings suggested that JTKY augmented acetoacetate and alpha-ketoglutaric acid levels in poly(I:C)-induced pneumonia model mice. Acetoacetate is a critical metabolite in the synthesis and degradation of ketone bodies, while alpha-ketoglutaric acid is a critical TCA cycle metabolite. Their possible association with viral pneumonia has been described under the “Synthesis and degradation of ketone bodies” and “TCA cycle” headings.

Our study has some limitations. Only male mice were used to evaluate the effects of JTKY in the poly(I:C)-induced pneumonia model. Further studies should be carried out to determine whether sex could affect the results. Additionally, a poly(I:C)-induced pneumonia model was used in this study, however, the relationship between poly(I:C)-induced pneumonia and COVID-19 requires further investigation. *In vitro* studies, such as the SARS-CoV-2 pseudovirus neutralization assay, can be carried out to further evaluate the antiviral effects of the major components in JTKY on SARS-CoV-2.

## Conclusion

**FIGURE 5:**
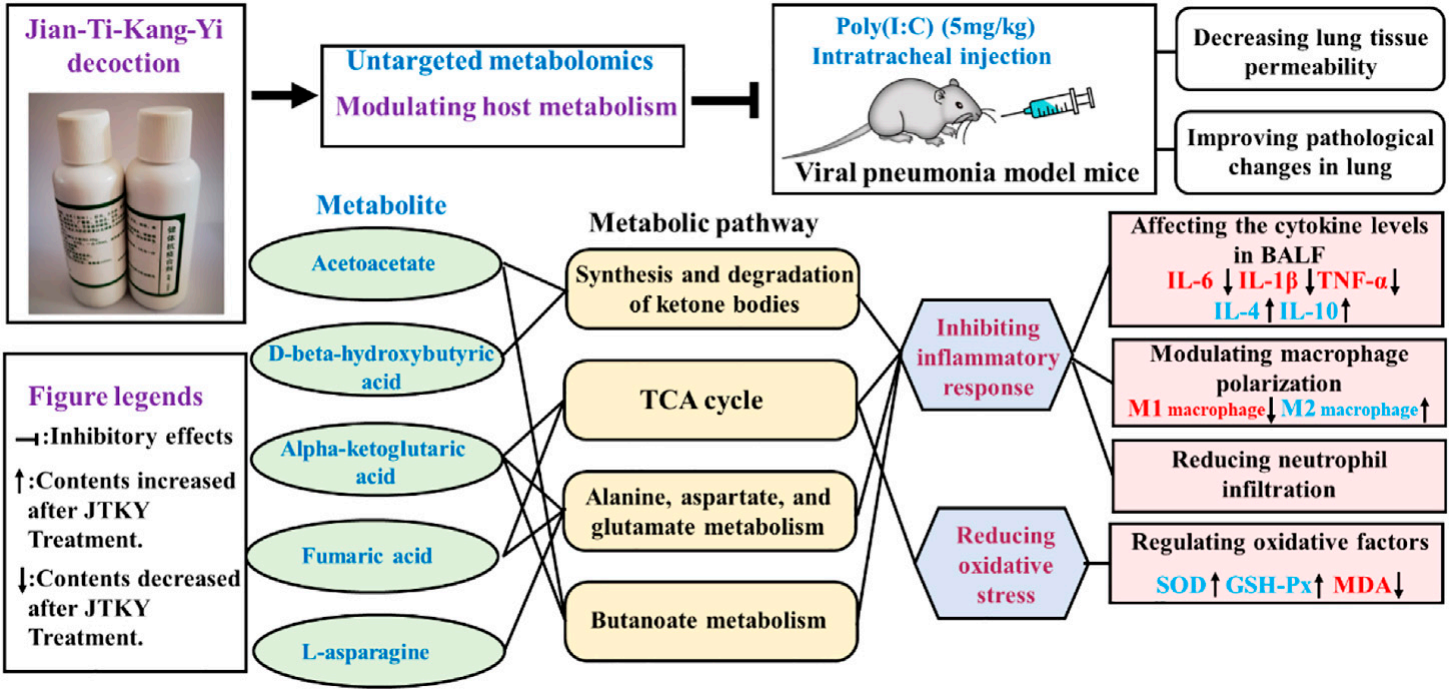
Graphical abstract. “┤” indicated the inhibitory effects. “↑” indicated the increase of contents after JTKY treatment. “↓” indicated the decrease of contents after JTKY treatment.

In conclusion, our study demonstrated that treatment with JTKY ameliorated poly(I:C)-induced pneumonia. The mechanism of action of JTKY in the treatment of pneumonia may be associated with the: inhibition of the inflammatory response, the reduction of oxidative stress, and the regulation of the synthesis and degradation of ketone bodies, regulation of the TCA cycle, and metabolism of alanine, aspartate, glutamate, and butanoate in lung (Figure 5).

## Data Availability

The original contributions presented in the study are included in the article/Supplementary Material, further inquiries can be directed to the corresponding authors.
