# Supplementary material for: Jian-Ti-Kang-Yi decoction alleviates poly(I:C)-induced pneumonia by inhibiting inflammatory response, reducing oxidative stress, and modulating host metabolism
Source: Front Pharmacol. 2022 Sep 6;13:979400. doi: 10.3389/fphar.2022.979400 (PMC9486163; doi:10.3389/fphar.2022.979400)
Supplement: Supplementary file 1 [file DataSheet1.docx]

**Experimental condition of ultra performance liquid chromatography (UPLC) coupled with quadrupole-time of-flight (Q-TOF) mass spectrometer (MS) systems**

Briefly, the test solution was injected onto an ACQUITY UPLC BEH C_18_ column (2.1mm×100mm, 1.7μm). The column temperature was 50°C. The injection volumn was 2 μL and the flow rate was 0.3 mL/min and. Mobile phase A was 0.1% formic acid aqueous solution and mobile phase B was acetonitrile contained 0.1% formic acid. The mobile phase conditions were: 0 min, 5% B; 1 min, 10% B; 6 min 60% B; 6.5 min 100% B; 10 min 100% B; 10.1 min 5% B; 13 min 5% B.

A Q-TOF MS equipped with an electrospray ionization (ESI) source was used for both positive and negative ionization scan modes (m/z ranges from 50 to 1,200 Da). The scan time was 0.2 s. The capillary voltages were 3,000 V (positive mode) and 2,200 V (negative mode) respectively. The desolvation temperature was 350°C and the source temperature was 100℃. The sample cone voltage was 40 V and the extraction cone voltage was 4V. The cone gas flow was 40 L/h and the desolvation gas flow was 800 L/h (both positive and negative modes).

**
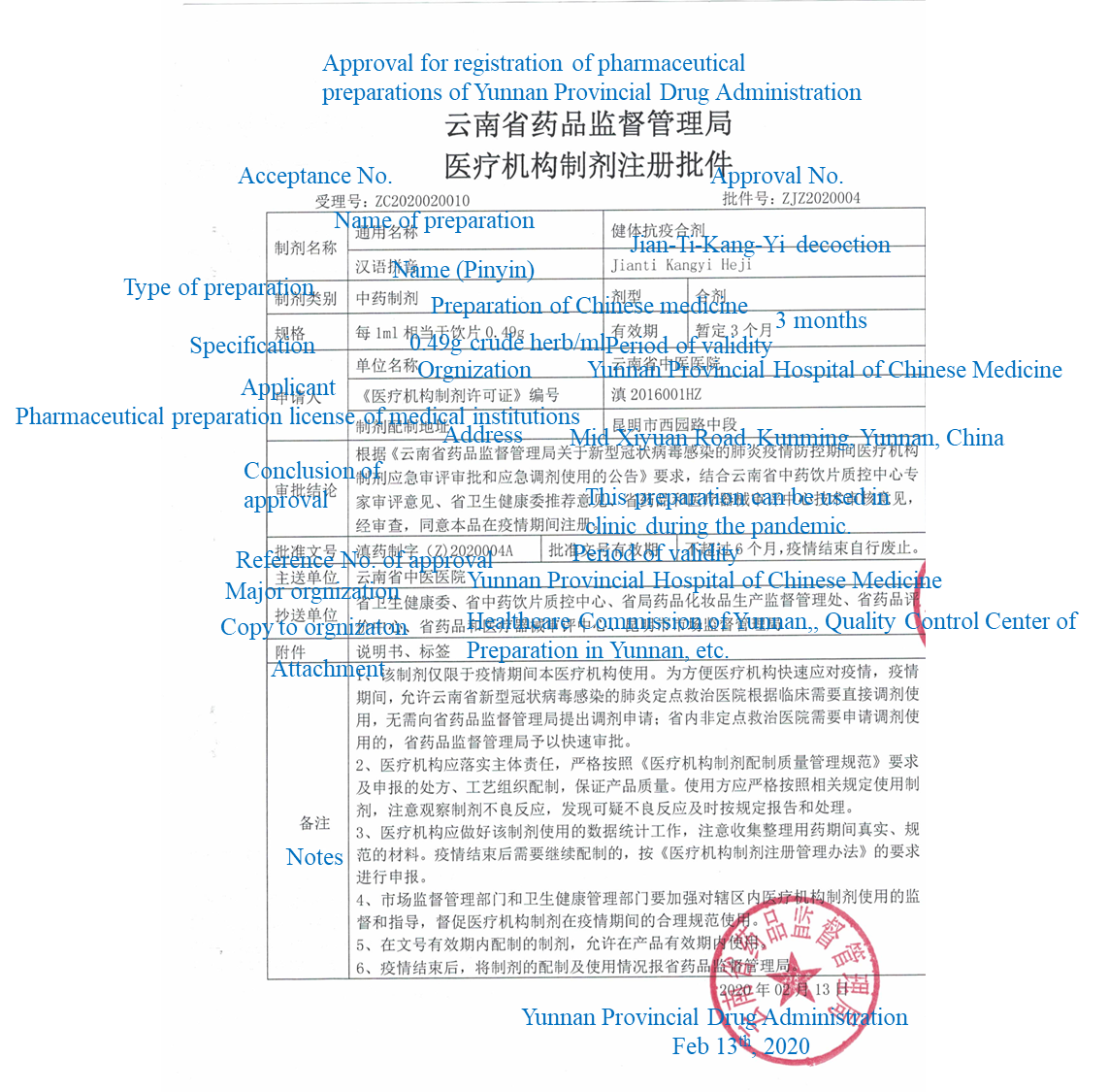
**

**FIGURE S1:** The production licence of JTKY


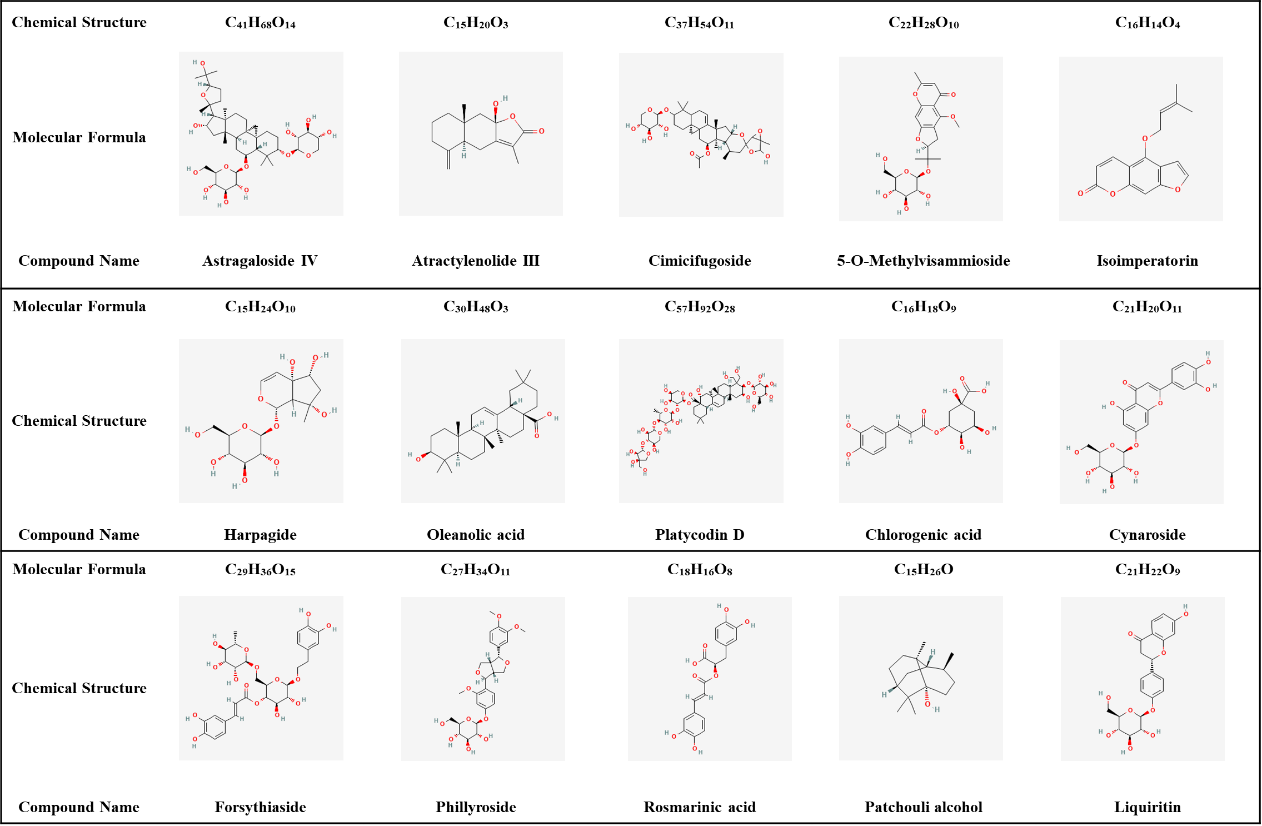


**FIGURE S2:** The molecular formulas and chemical structures of reference standards.

a


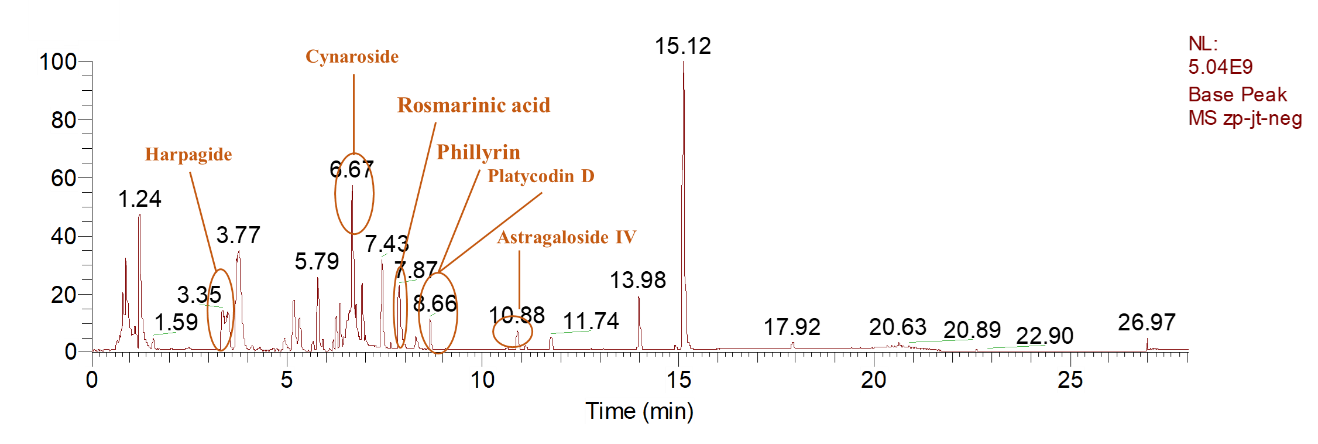


b


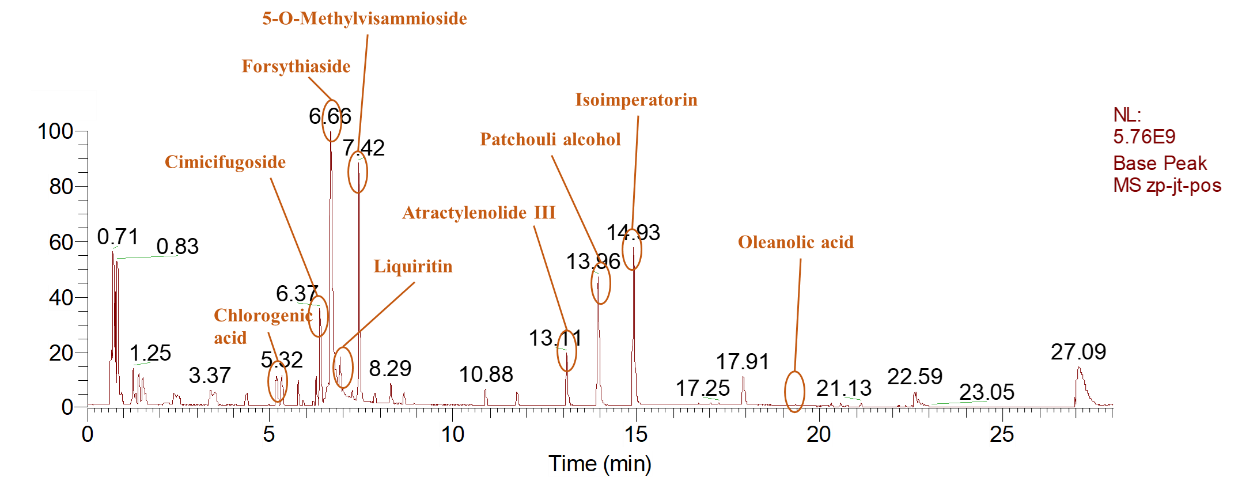


c


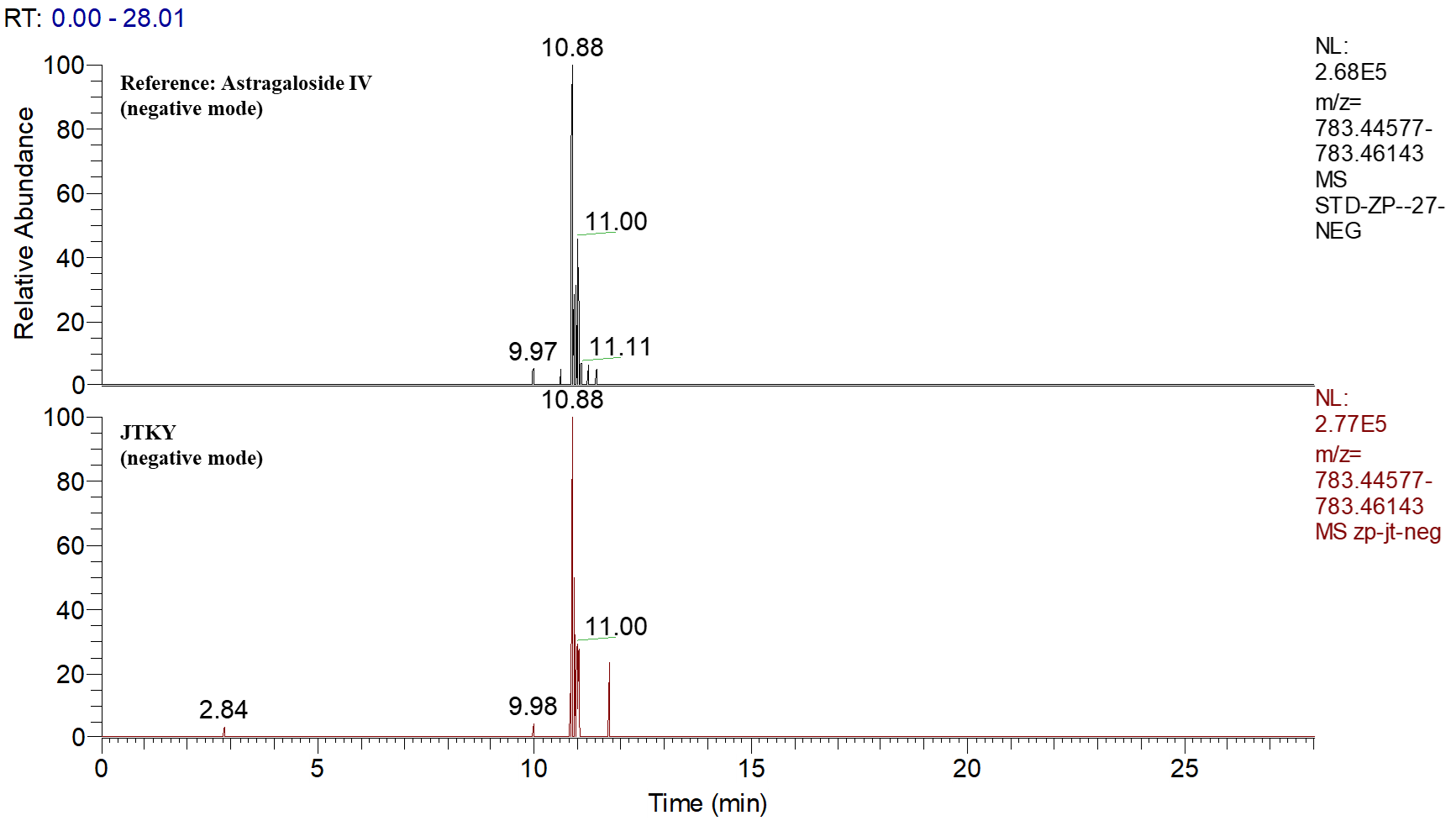


d


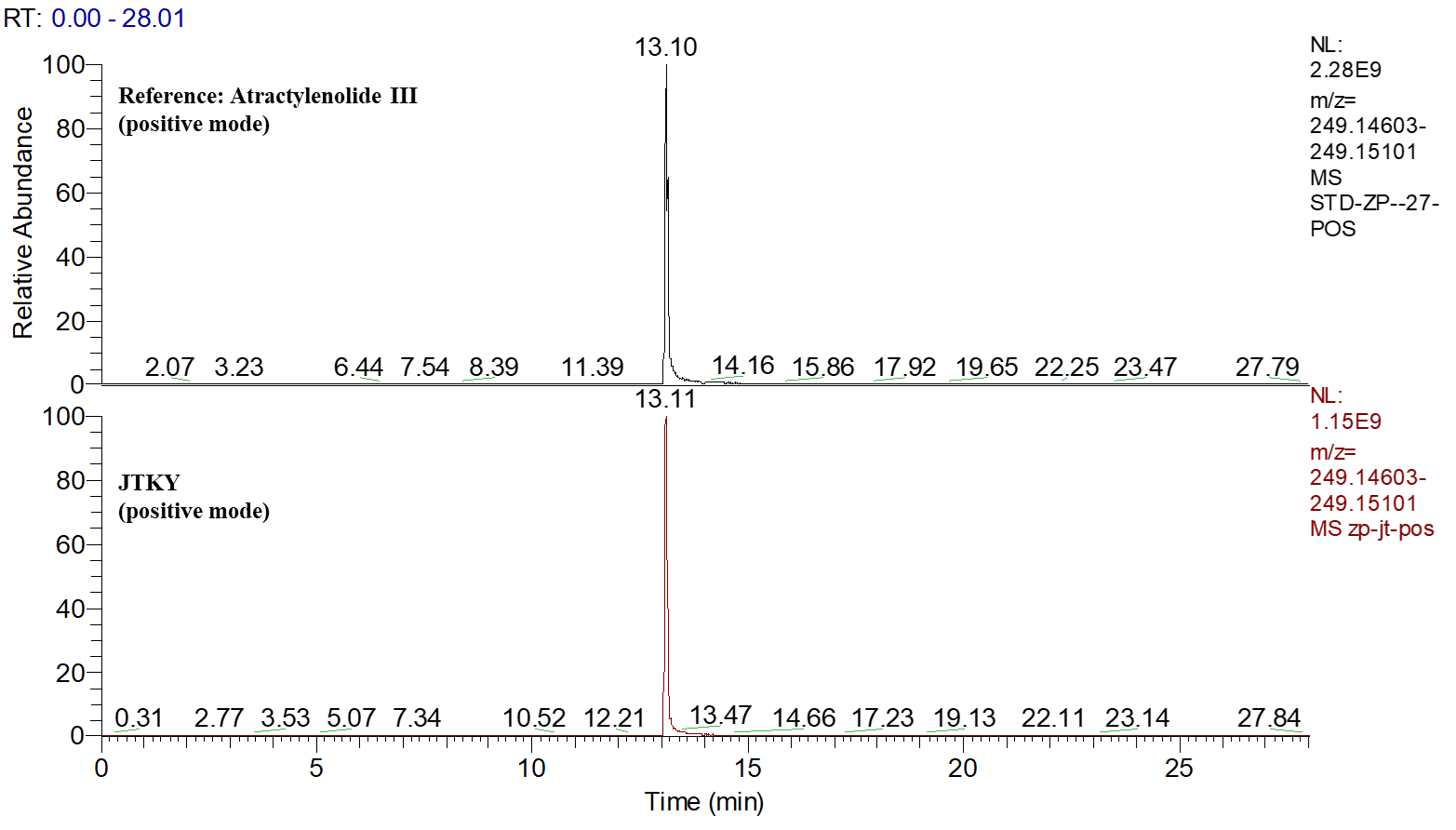


e


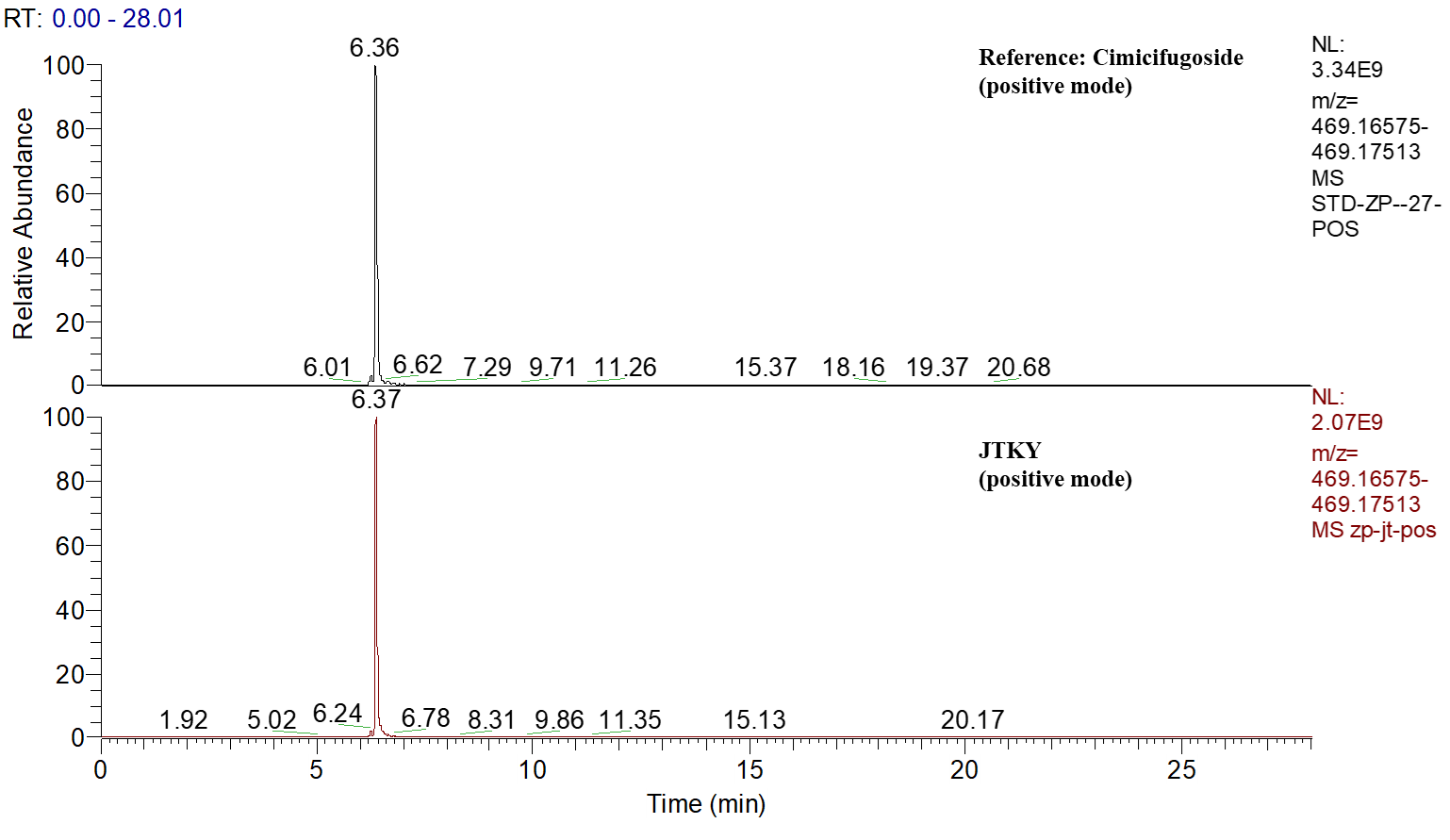


f


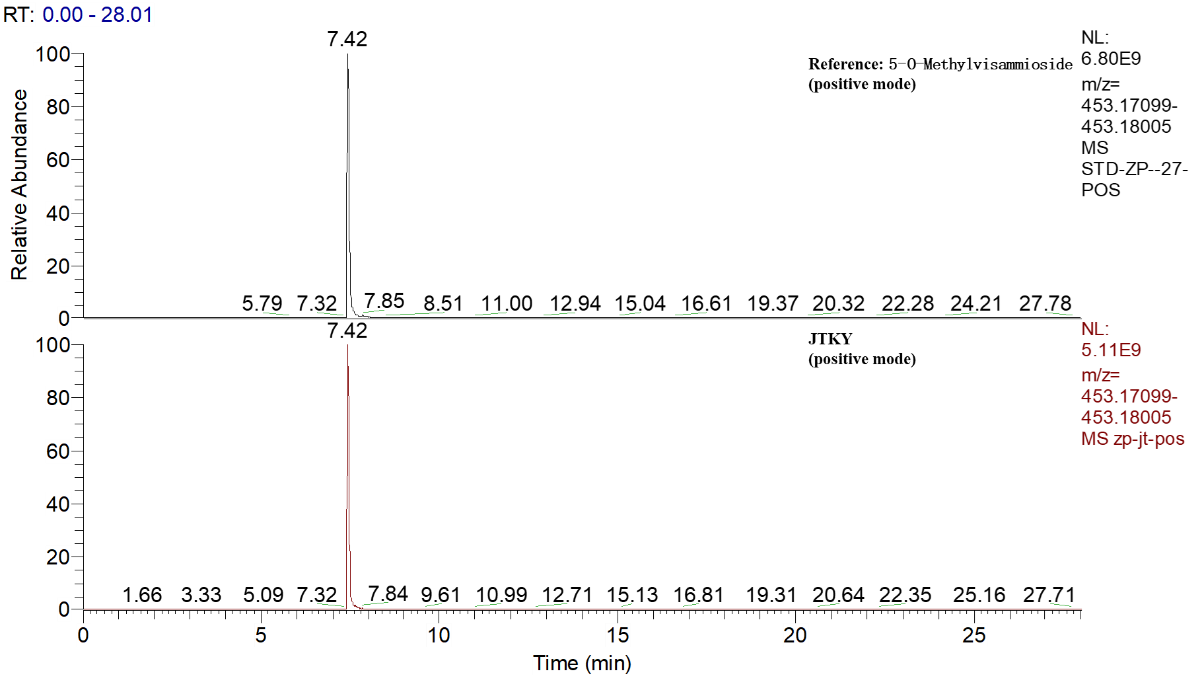


g


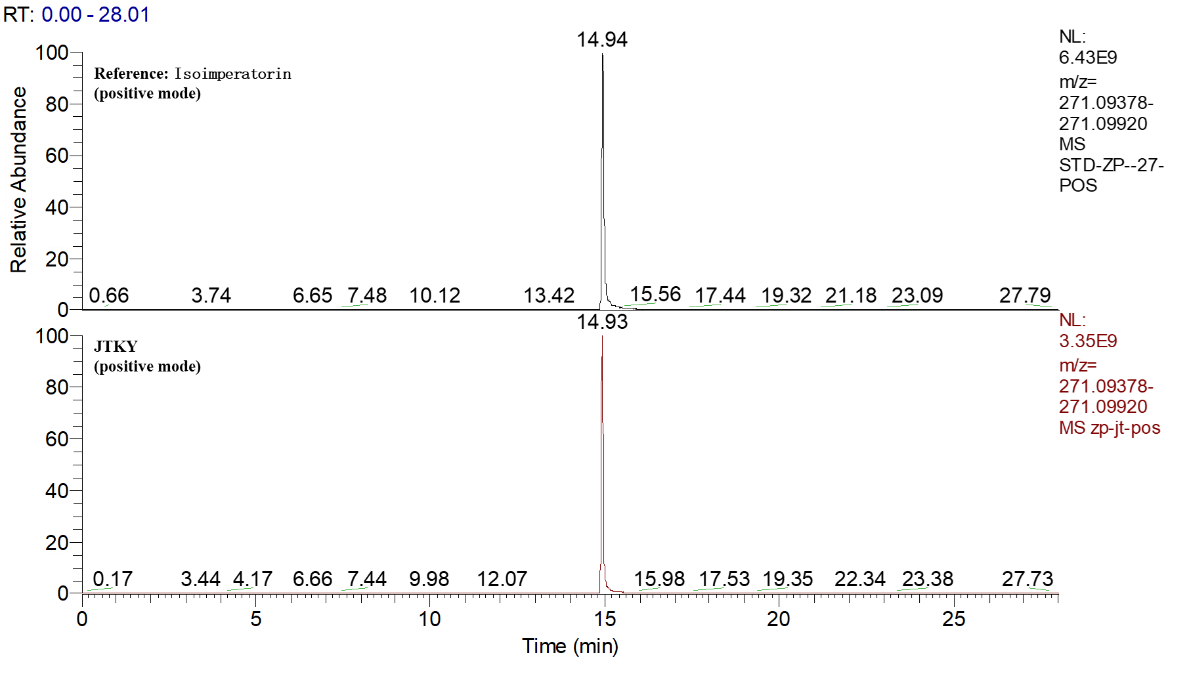


h


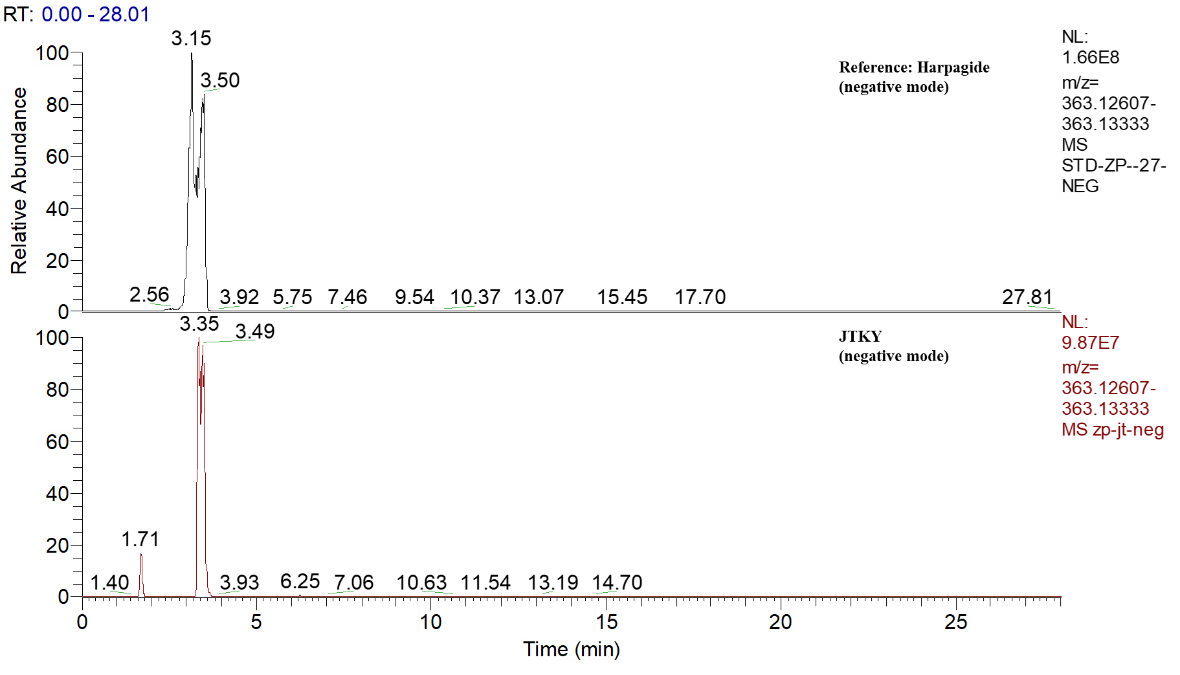


i


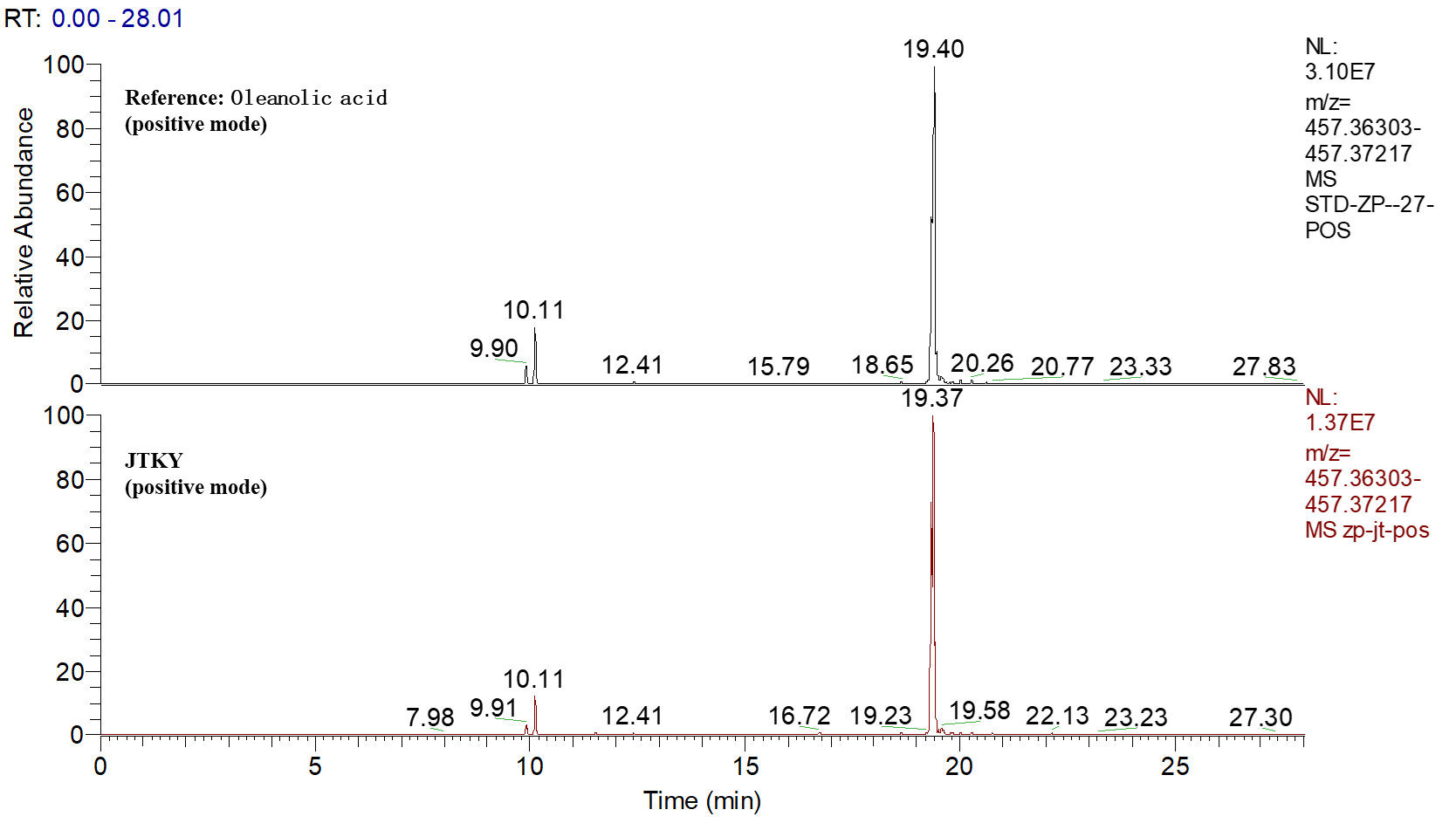


j


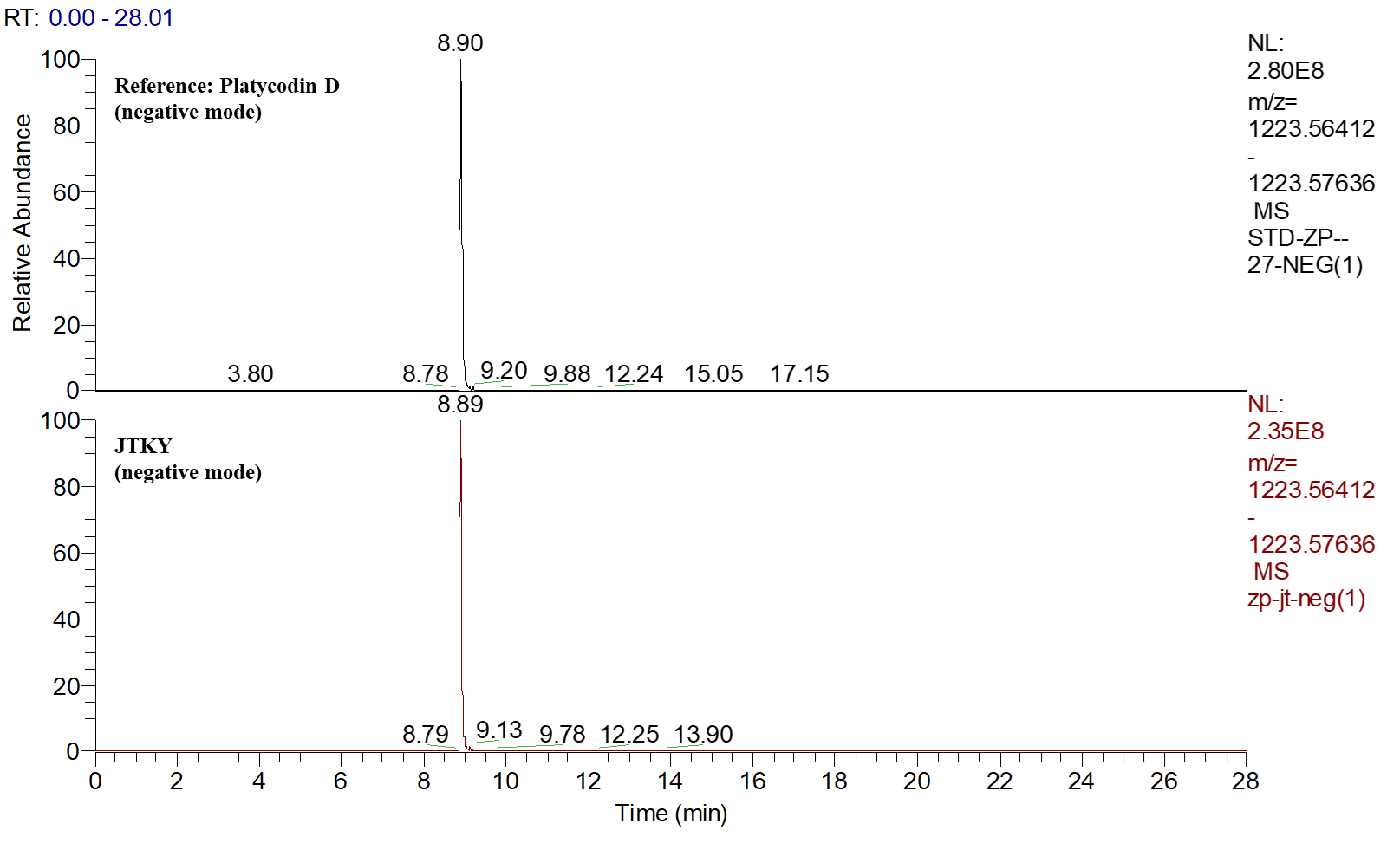


k


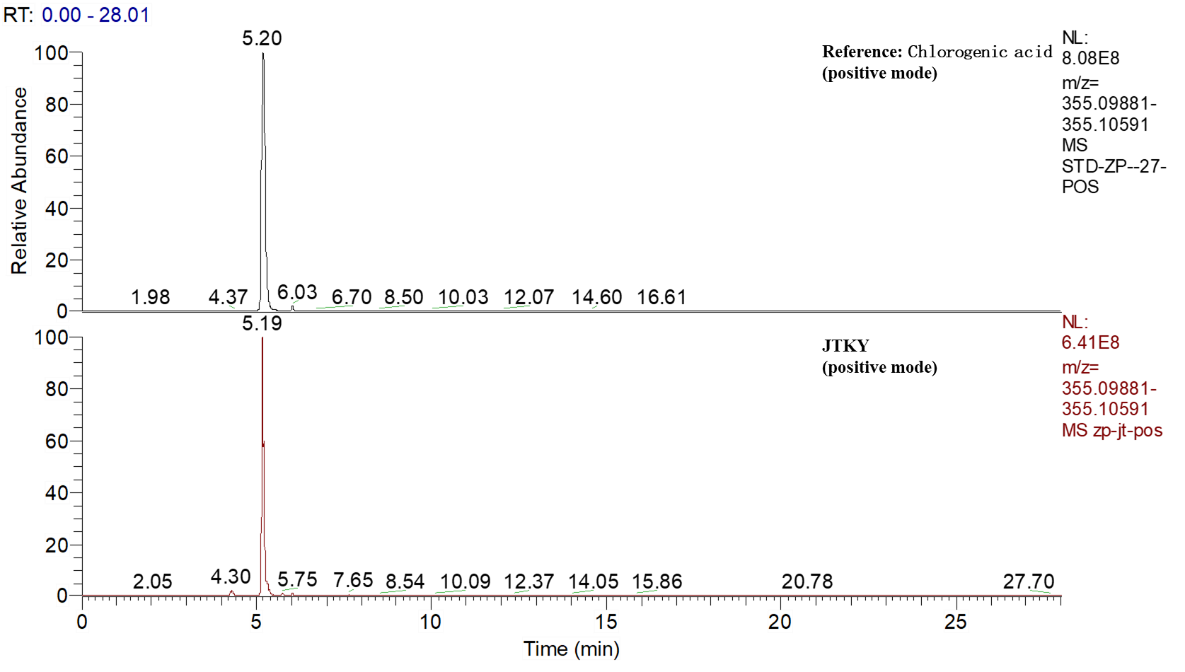


l


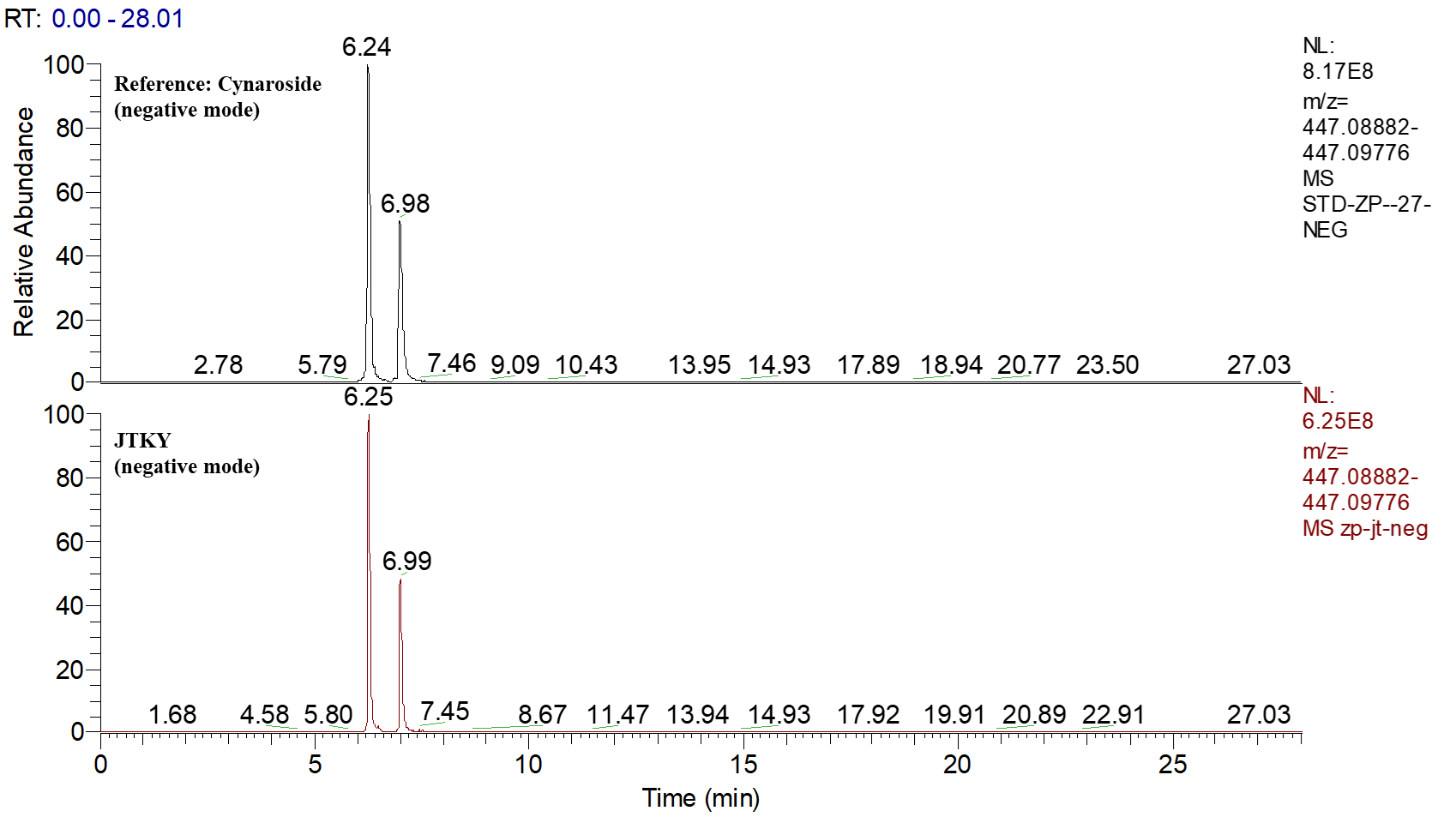


m


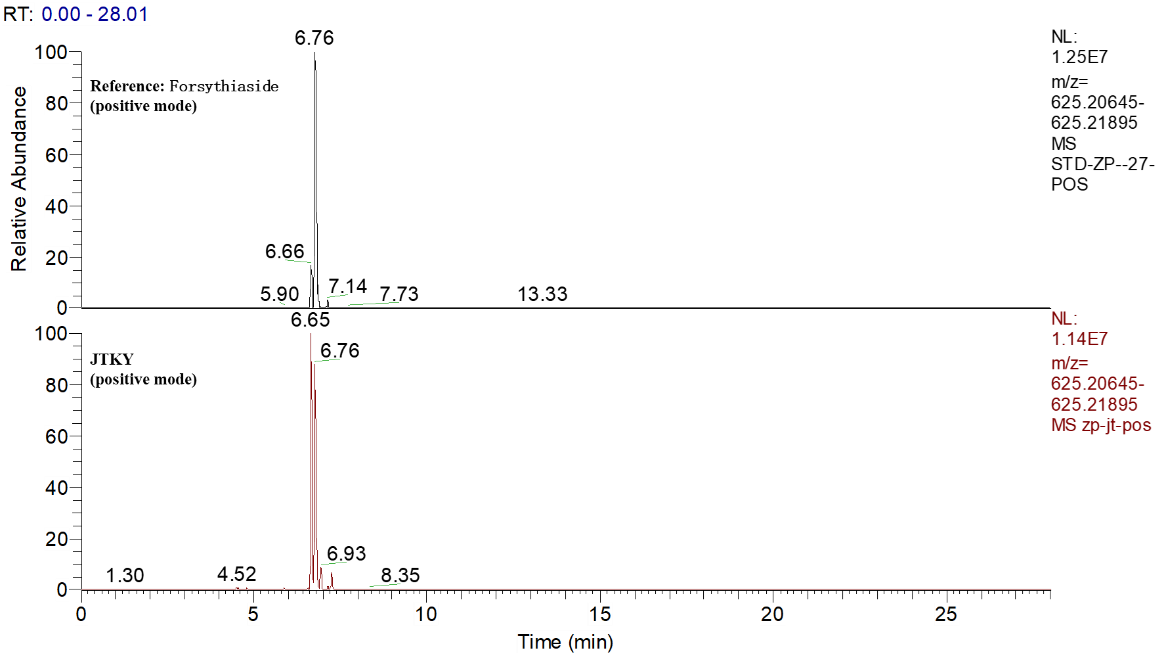


n


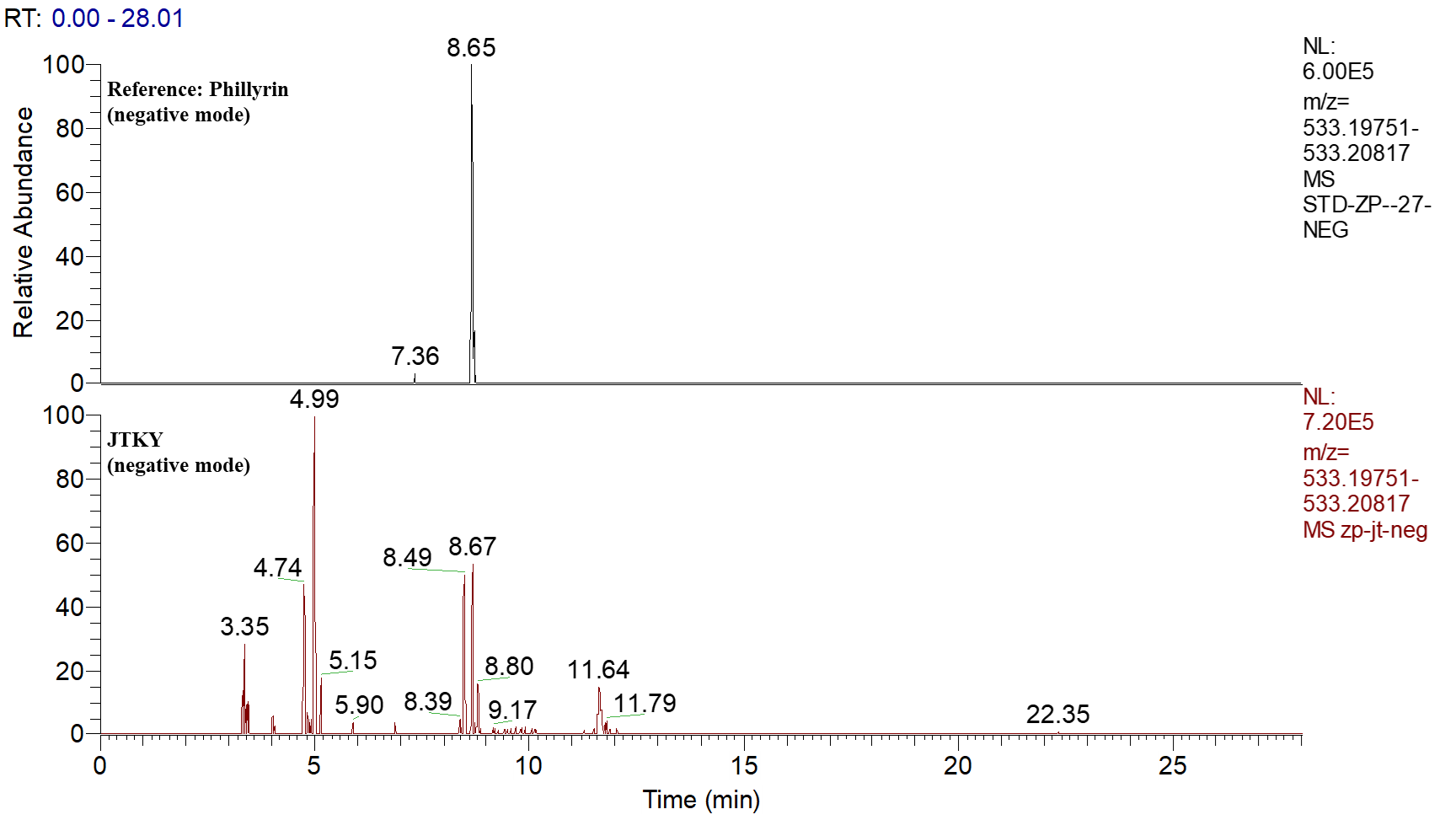


o


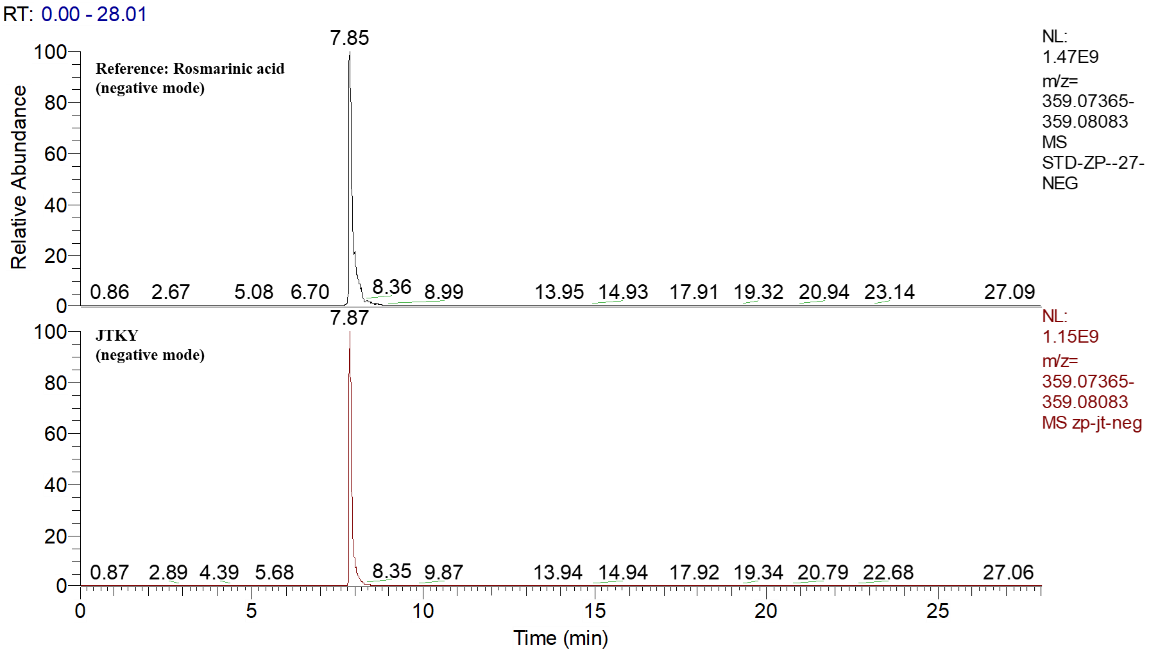


p


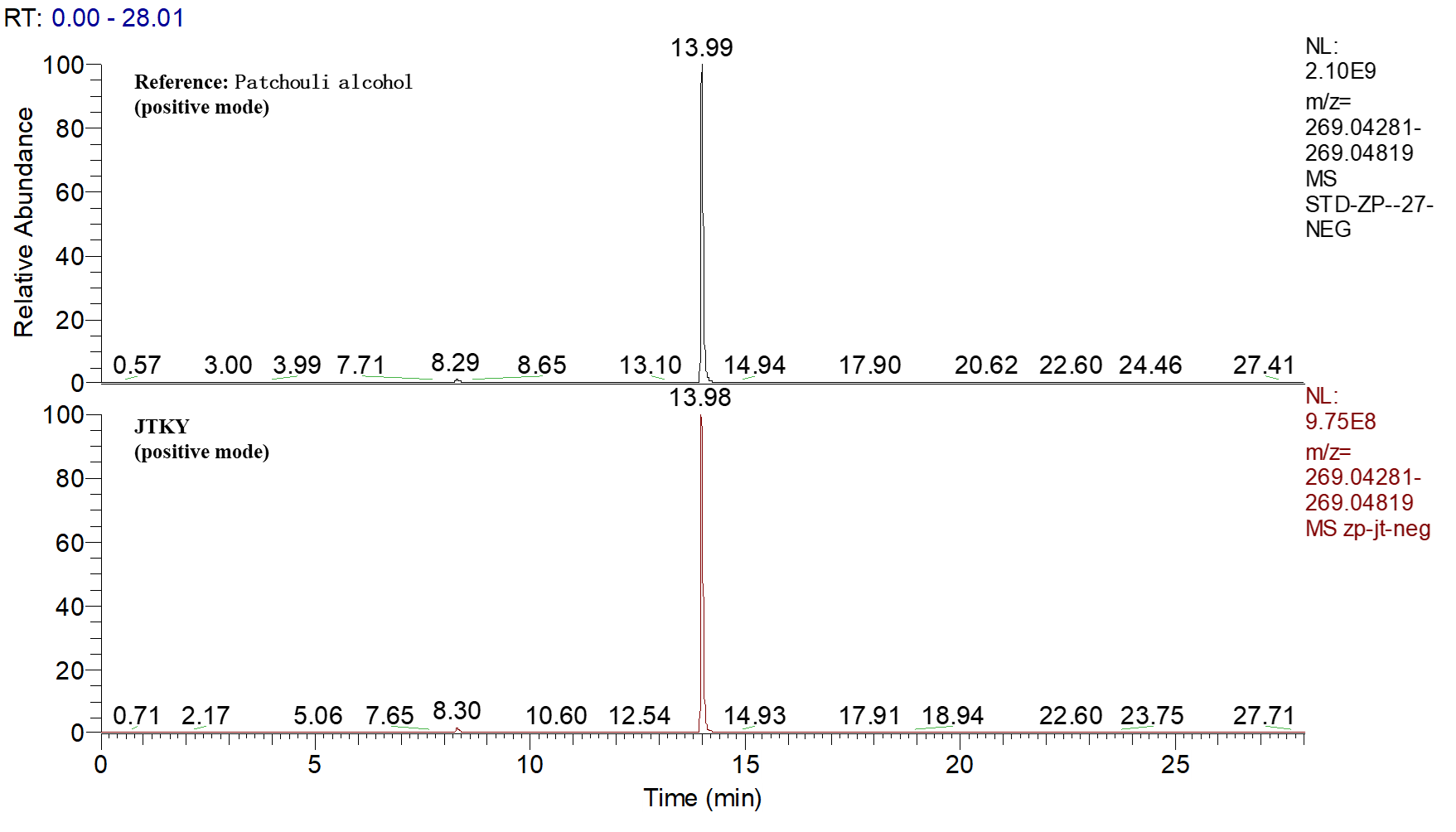


q


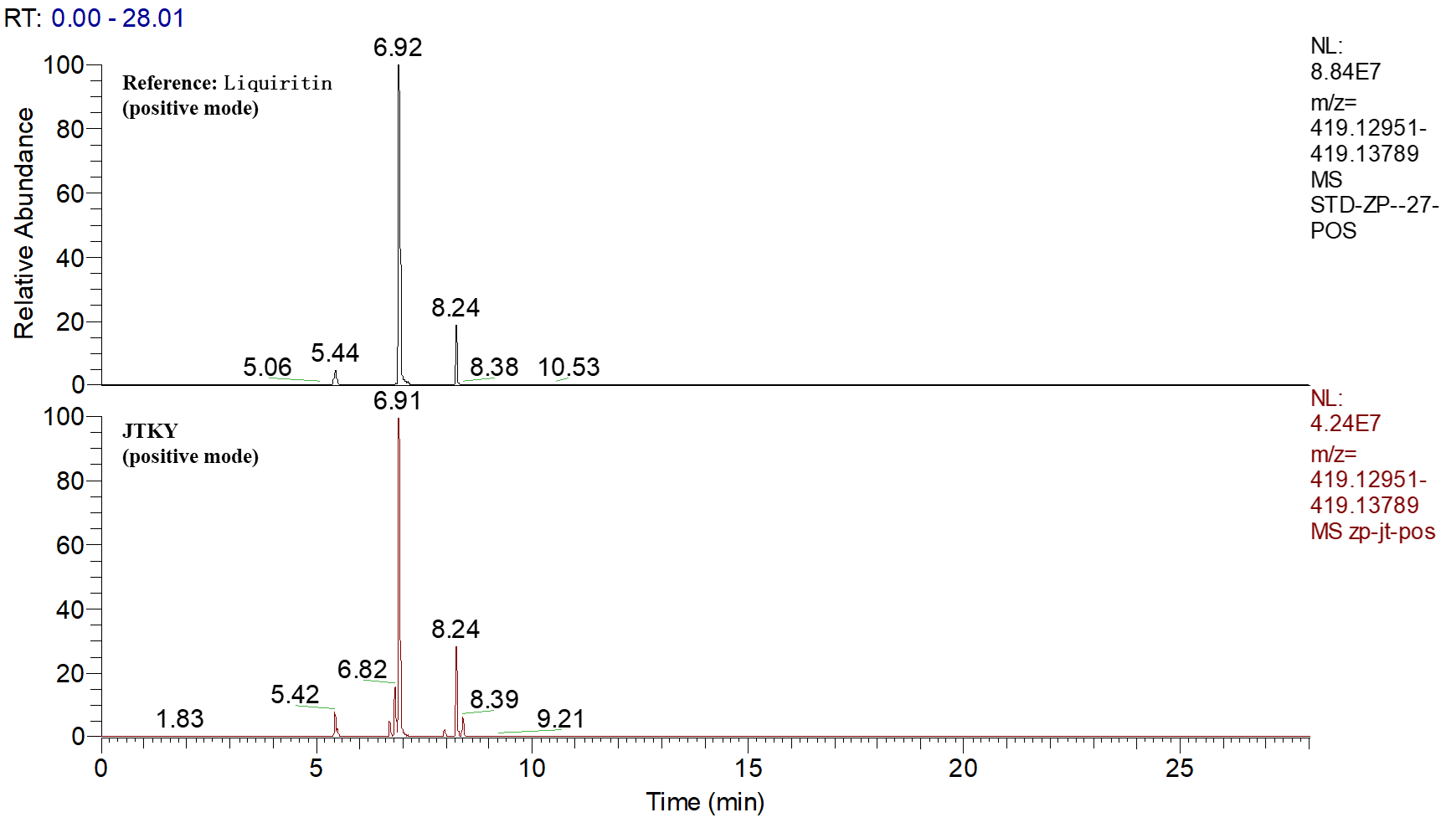


**Figure S3:** The chemical profiles of JTKY using UPLC-MS. (**a, b**) The total ion chromatogram in negative (**a**) and positive ion modes (**b**). (**c-q**) The main bioactive compounds of Astragaloside IV (c), Atractylenolide III (d), Cimicifugoside (e), 5-O-Methylvisammioside (f), Isoimperatorin (g), Harpagide (h), Oleanolic acid (i), Platycodin D (j), Chlorogenic acid(k), Cynaroside (l), Forsythiaside (m), Phillyrin (n), Rosmarinic acid (o), Patchouli alcohol (p), Liquiritin (q).

**TABLE S1 HE satining score for lung injury** (Cui et al., 2020)

| **Score** | **Description** |
| --- | --- |
| 0 (None) | No inflammatory cells can be detected. |
| 1 (Mild) | Thickening of the alveolar septum by a mononuclear cell infiltrate, with involvement limited to focal, pleural-based lesions occupying less than 20 % of the lung and with good preservation of the alveolar architecture |
| 2 (Moderate) | A more widespread alveolitis involving 20 to 50 % of the lung, although still predominantly pleural based |
| 3 (Severe) | A diffuse alveolitis involving more than 50 % of the lung, with occasional consolidation of air spaces by the intra-alveolar mononuclear cells and some hemorrhagic areas within the interstitium and /or alveolus |

**TABLE S2 Masson staining score** (Zhou et al., 2016)

| **Score** | **Description** |
| --- | --- |
| 0 (-) | No pulmonary fibrosis. |
| 1 (+) | Light degree of pulmonary fibrosis,  lesion range <20% in the whole lung. |
| 2 (++) | Moderate pulmonary fibrosis, lesion range ~20–50% in the whole lung. |
| 3 (+++) | Severe pulmonary fibrosis, lesion range >50% in the whole lung, accompanied by alveolar fusion and lung parenchyma structural disorder. |

**TABLE S3 The characteristic fragment ions of reference standards in JTKY**

| **Marking**  **peak no.** | **Name** | **RT**  **(min)** | **Ion** |
| --- | --- | --- | --- |
| 1 | Astragaloside IV | 10.88 | [M-H]^-^ |
| 2 | Atractylenolide III | 13.10 | [M+H]^+^ |
| 3 | Cimicifugoside | 6.36 | [M+H]^+^ |
| 4 | 5-O-Methylvisammioside | 7.42 | [M+H]^+^ |
| 5 | Isoimperatorin | 14.94 | [M+H]^+^ |
| 6 | Harpagide | 3.50 | [M-H]^-^ |
| 7 | Oleanolic acid | 19.40 | [M+H]^+^ |
| 8 | Platycodin D | 8.90 | [M-H]^-^ |
| 9 | Chlorogenic acid | 5.20 | [M+H]^+^ |
| 10 | Cynaroside | 6.98 | [M-H]^-^ |
| 11 | Forsythiaside | 6.76 | [M+H]^+^ |
| 12 | Phillyrin | 8.65 | [M-H]^-^ |
| 13 | Rosmarinic acid | 7.85 | [M-H]^-^ |
| 14 | Patchouli alcohol | 13.99 | [M+H]^+^ |
| 15 | Liquiritin | 6.92 | [M+H]^+^ |
